# AIM2 enhances *Candida albicans* infection through promoting macrophage apoptosis via AKT signaling

**DOI:** 10.1007/s00018-024-05326-9

**Published:** 2024-06-25

**Authors:** Qian Jiang, Yayun Chen, Siping Zheng, Lina Sui, Dalang Yu, Furong Qing, Wenji He, Qiuxiang Xiao, Tianfu Guo, Li Xu, Zhichun Liu, Zhiping Liu

**Affiliations:** 1 School of Graduate, China Medical University, Shenyang, Liaoning, China; 2 School of Nursing, Gannan Medical University, Ganzhou, Jiangxi China; 3 School of Basic Medicine, Gannan Medical University, Ganzhou, Jiangxi China; 4 School of Graduate, Gannan Medical University, Ganzhou, Jiangxi China; 5 Department of Pathology, The First Affiliated Hospital of Gannan Medical University, Ganzhou, Jiangxi China; 6 Center for Scientific Research, Gannan Medical University, Ganzhou, Jiangxi China; 7 Center for Immunology, Key Laboratory of Prevention and Treatment of Cardiovascular and Cerebrovascular Diseases, Ministry of Education, Gannan Medical University, Ganzhou, Jiangxi China

**Keywords:** AIM2, *Candida albicans*, Macrophage, Apoptosis, AKT

## Abstract

*Candida albicans* is among the most prevalent invasive fungal pathogens for immunocompromised individuals and novel therapeutic approaches that involve immune response modulation are imperative. Absent in melanoma 2 (AIM2), a pattern recognition receptor for DNA sensing, is well recognized for its involvement in inflammasome formation and its crucial role in safeguarding the host against various pathogenic infections. However, the role of AIM2 in host defense against *C. albicans* infection remains uncertain. This study reveals that the gene expression of AIM2 is induced in human and mouse innate immune cells or tissues after *C. albicans* infection. Furthermore, compared to their wild-type (WT) counterparts, *Aim2*^*−/−*^ mice surprisingly exhibit resistance to *C. albicans* infection, along with reduced inflammation in the kidneys post-infection. The resistance of *Aim2*^*−/−*^ mice to *C. albicans* infection is not reliant on inflammasome or type I interferon production. Instead, *Aim2*^*−/−*^ mice display lower levels of apoptosis in kidney tissues following infection than WT mice. The deficiency of AIM2 in macrophages, but not in dendritic cells, results in a phenocopy of the resistance observed in *Aim2*^*−/−*^ mice against *C. albican* infection. The treatment of Clodronate Liposome, a reagent that depletes macrophages, also shows the critical role of macrophages in host defense against *C. albican* infection in *Aim2*^*−/−*^ mice. Furthermore, the reduction in apoptosis is observed in *Aim2*^*−/−*^ mouse macrophages following infection or treatment of DNA from *C. albicans* in comparison with controls. Additionally, higher levels of AKT activation are observed in *Aim2*^*−/−*^ mice, and treatment with an AKT inhibitor reverses the host resistance to *C. albicans* infection. The findings collectively demonstrate that AIM2 exerts a negative regulatory effect on AKT activation and enhances macrophage apoptosis, ultimately compromising host defense against *C. albicans* infection. This suggests that AIM2 and AKT may represent promising therapeutic targets for the management of fungal infections.

**Supplementary Information:**

The online version contains supplementary material available at 10.1007/s00018-024-05326-9.

## Introduction

Invasive fungal infections are responsible for a significant number of fatalities globally, with *C. albicans* being a prevalent pathogenic fungus in humans [1]. An increasing number of immunodeficient individuals, such as those infected with HIV, recipients of organ transplants, and cancer patients undergoing chemotherapy, are susceptible to invasive fungal infections. The limited availability of antifungal drugs in clinical settings and frequent occurrence of drug resistance contribute to a high incidence and mortality rate of invasive fungal-related diseases [2]. Therefore, understanding on the immune system’s response to fungal infections is crucial for the advancement of novel immunotherapies [3, 4].

The recognition of *C. albicans* involves the activation of a signal pathway primarily mediated by pattern recognition receptors (PRRs) [5]. PRRs are innate immune cell receptors that are encoded by the germline and capable of recognizing one or more pathogen-associated molecular patterns (PAMPs) or damage-associated molecular patterns (DAMPs). These receptors encompass Toll-like receptors (TLRs), Nod-like receptors (NLRs), RIG-I-like receptors (RLRs), Aim2-like receptors (ALRs), and C-type lectin-like receptors (CLRs) [6].

Prior research has identified TLRs and CLRs as the primary PRRs involved in the recognition of *C. albicans*. TLR2 and TLR4 are commonly recognized as the primary receptors responsible for the identification of *C. albicans* among TLRs [7]. Upon activation of TLRs by PAMPs originating from the surface of *C. albicans*, TIR is activated and binds to the adaptor protein MyD88 or TRIF, thereby initiating the downstream protein kinase cascade. This, in turn, leads to the nuclear translocation of transcription factors such as NF-κB and IRF3/7, ultimately resulting in the production of pro-inflammatory cytokines and type I interferon [7]. The binding of fungal cell wall components to Dectin-1/2 in CLRs results in the activation of Syk and CARD 9 pathways, leading to the production of cytokines and chemokines that are crucial in the elimination of extracellular fungus [8]. However, unstrained recruitment of inflammatory cells to the kidneys, the primary target organ of invasive *candidiasis*, and excessive production of cytokine or chemokines may trigger renal immunopathology, leading to sepsis or death [9–12].

Nonetheless, subsequent investigations have revealed the involvement of other PRRs in the immune response to *C. albicans* infection. For instance, Nod-like receptors NLRP3 and NLRC4 activate inflammasome, leading to IL-1β production, and stimulate the production of antimicrobial peptides to counteract *C. albicans* infection [13, 14].

The protein Absent in melanoma 2 (AIM2) functions as a PRR that recognizes double stranded DNA (dsDNA) and is classified as a member of the ALR family. AIM2 is located in the cytoplasm or nucleus and is composed of a HIN200 domain, which binds dsDNA, and a PYD domain, which transmits intracellular signals. AIM2 forms an inflammasome complex with the adaptor protein ASC and protease caspase 1, which is responsible for the cleavage of IL-1β and IL-18 precursors into mature and secretory forms, and ultimately mediates pyroptosis, a form of programmed cell death [15].

There is a wealth of evidence supporting the recognition of foreign pathogen dsDNA by AIM2, which subsequently activates inflammasome and plays a crucial role in host resistance against viral and intracellular bacterial infections [16–20]. However, AIM2 also recognizes dsDNA released by host cells and can contribute to the development of aseptic inflammatory diseases, such as ischemic brain damage, diabetes, atherosclerosis, and chronic nephritis, by stimulating the production and release of inflammatory cytokines [21–24]. Additionally, AIM2 exhibits a multifaceted regulatory role in various cancers [25, 26]. Prior research has demonstrated that AIM2 can facilitate the development of skin squamous cell carcinoma and non-small cell lung cancer, while impeding the onset of hepatocellular carcinoma [27–30]. Our previous investigation unveiled the intricate mechanism by which AIM2 inhibits colorectal cancer by regulating intestinal stem cell activity and inducing changes in gut microbiota [31]. These fingings imply that AIM2 may exhibit diverse functions in various diseases.

Despite the primary function of AIM2 in mediating inflammasome activation, our research and others have also identified its ability to operate autonomously from inflammasome activation [31, 32]. In this instance, AIM2 impeded the proliferation of intestinal epithelial cells by repressing the Akt signaling pathway [31, 32]. Additional research has demonstrated that AIM2 can inhibit the Akt signaling pathway in an inflammasome-independent manner, thereby enhancing intestinal epithelial integrity and augmenting the host’s resistance to *Salmonella* infection [33]. These findings indicate that AIM2 can function through either an inflammasome-dependent or inflammasome-independent mechanism.

Currently, there is a paucity of research on the role of AIM2 in fungal infections. A study has demonstrated that the AIM2 inflammasome, in collaboration with the NLRP3 inflammasome, enhances host resistance to *Aspergillus fumigatus* infection, but not independently [34]. However, the role of AIM2 in *C. albican*s infection remains unexplored, and its mechanism in relation to inflammasome is unclear.

In this study, we show that the gene expression of AIM2 is induced in human and mouse innate immune cells or tissues after *C. albicans* infection. The absence of AIM2 provides protection against systemic *C. albicans* infection in mice, and this protection is not associated with the inflammasome or interferon pathway. Notably, the findings suggest that *AIM2*^*−/−*^ may mitigate *C. albicans* infection by reducing apoptosis through Akt activation. In conclusion, our study elucidates the immune recognition of fungal pathogens by the host innate immune receptors and presents novel therapeutic approaches for fungal infections.

## Materials and methods

### Clinical data

To assess the association between AIM2 transcriptional induction and healthy human cells stimulated with *C. albicans*, previously published public transcriptional data were sourced, as deposited under the accession number GSE69723, GSE42606 and GSE162746. All RNA-seq data had undergone strict quality control, which mainly included the following preprocessing steps, background correction, normalization, PM-correction, and summarization.

### Mice

Male C57BL/6 mice aged 8–10 weeks were procured from Nanjing Model Animal Center (Nanjing, Jiangsu, China) as the wild type (WT) group. *Aim2*^*−/−*^ mice were generously provided by Xiaopeng Qi from the Kunming Institute of Zoology, Chinese Academy of Sciences. Yinming Liang from Xinxiang Medical University, Henan, China, provided A*im2*^*fl/fl*^ mice. *Asc*^*−/−*^ mice, *CD11c-cre* mice, and *Lyz-cre* mice were obtained from Cyagen Biotechnology Company (Suzhou, Jiangsu, China). *Aim2*^*fl/fl*^*; CD11c-cre* and *Aim2*^*fl/fl*^*; Lyz-cre* mice were generated through breeding *Aim2*^*fl/fl*^ mice with *CD11c-cre* and *Lyz-cre* mice, respectively. The mice were housed in a specific pathogen-free facility and all animal research was approved by the Ethics Committee of Gannan Medical University (Ganzhou, Jiangxi, China).

### Fungal preparation

*Candida albicans* (#SC5314), obtained from Dr. Changbin Chen (Shanghai Institute of Immunity and Infection, Chinese Academy of Sciences, Shanghai, China), was utilized for all in vivo and in vitro studies. *C. albicans* were cultured according to previously described methods with modifications [35]. *C albicans* were cultured in YPD　liquid　medium overnight and then recultured the next day to log phase, after which they were washed twice with 1 × PBS and resuspended in sterile PBS. Prior to infection, the *C. albicans* cells were counted using a Neubauer chamber.

### RT-qPCR

RT-qPCR assays were utilized to determine the expression levels of genes. Total RNA was extracted from tissues or cells using TRIzol (Invitrogen) and subsequently reverse transcribed into cDNA. Gene expression levels were quantified using a fully automatic fluorescence quantitative PCR System (SLAN-96P, China) with 20 ng cDNA as a template. β-actin was employed for normalization purposes. The relative gene expression level was calculated using the 2^*− ΔΔCt*^ method. Supplementary Table 1 provides a comprehensive list of all primers used in this study.

### *C. albicans* systemic infection

*C. albicans* yeast was administered intravenously to mice at a concentration of 2 × 10^5^ fungi per mouse in a 100 uL volume. The infected mice were subjected to daily monitoring for weight loss and survival. To determine the fungal burden, the mice were humanely sacrificed, and their kidneys were aseptically removed, weighed, homogenized, serially diluted, and plated onto YPD agar plates. The fungal colony-forming units (CFUs) were determined after incubation at 30 °C for 24 h, and the fungal burdens were expressed as CFUs per gram of tissue.

### Histopathology analysis

The tissues were subjected to histopathology analysis by fixing them in 4% paraformaldehyde, embedding them in paraffin, and sectioning them into 5 μm thick sections. These sections were then stained with haematoxylin–eosin (H&E), periodic-acid-Schiff (PAS) and subsequently scanned using a Panoramic histiocyte quantitative analysis system (TissueFAXS Plus, TissueGnostics) to assess the severity of inflammation and intra-lesional fungal burden. The renal inflammation score was determined according to previously established protocols [36]. Briefly, renal inflammation was scored based on H&E stainings (Proportion of renal parenchyma and/or pelvis involved by tubulointerstitial nephritis and/or pyelonephritis). Inflammatory lesions area (%) = Inflammatory focus area/ the entire kidney area * 100. Then, the scoring system utilized assigned 0 points for no inflammatory lesions, 1 point for inflammatory lesions less than 10%, 2 points for 10–25%, 3 points for 25–50%, and 4 points for greater than 50%.

### ELISA analysis

Cytokines in sera or tissue homogenate were quantified using ELISA kits and following the manufacturer’s instructions. The diluted protein samples were incubated at 37 °C for 1 h on ELISA plates. Following the washing step, the enzyme-labeled antibody was introduced, following by the addition of the substrate solution to facilitate color development. The reaction was terminated, and the optical density (OD) values were measured at 450 nm. All ELISA kits were procured from Jingmei Biotechnology Co., Ltd (Jiangsu, China), and their information were provided in Supplementary Table 2.

### Western-blot

Protein extraction from tissues or cells was performed using RIPA lysate supplemented with protease/phosphatase inhibitors, and the protein concentration was quantified using the BCA kit (# 23,227, Thermo Scientific). The protein samples were subjected to SDS-PAGE and transferred onto a PVDF membrane (IPVH00010, Millipore). The blots were blocked with 5% bovine serum albumin, and subsequently incubated with primary antibodies overnight at 4 °C. Following washing, goat HRP-conjugated secondary antibodies were introduced. Immunoreactivity was detected through ECL chemical fluorescence chromogenic solution (#34096, Thermo Scientific) and a BIO-RAD ChemDoc (BIO-RAD, USA). Densitometry analysis was performed using ImageJ software (NIH, USA). The pertinent information for the primary antibodies were shown in Supplementary Table 3.

### Cell culture

Murine bone marrow-derived macrophages (BMDM) were cultured according to previously established protocols [37]. Specifically, the bone marrow of femur and tibia were cultured in IMDM medium supplemented with 1% L-glutamine, 10% fetal bovine sera (Hyclone), 1% penicillin–streptomycin, and 30% L929 cell culture supernatant. Following the removal of nonadherent cells, over 90% cells are positive for the macrophage marker CD11b^+^ F4/80^+^as determined by FACS analysis (Figure S1A). After 6 days of incubation, BMDMs were plated into 12-well plates at a density of 1 × 10^6^/well for infection assays.

Bone marrow-derived dendritic cells (BMDCs) were prepared according to previously protocols [34]. In brief, bone marrow cells were grown in RPMI1640 supplemented with 10% FBS, 1% penicillin–streptomycin, 1% non-essential amino acid, 1% sodium pyruvate and 20 ng/mL GM-CSF for 7 days. Over 90% of cells are positive for the dendritic cell marker CD11b^+^CD11c^+^ as determined by FACS analysis (Figure S1B). BMDCs (1 × 10^6^) were seeded in 12-well cell culture plates.

### Collection of peritoneal macrophages

Cells were harvested by flushing the peritoneal lavage with 5 mL of cold sterile PBS solution and then erythrocytes were lysed. The cells were plated in culture medium (RPMI 1640 supplemented with 10% FBS, 1% Penicillin–Streptomycin solution).

### Flow cytometry

The single-cell suspensions of BMDMs/BMDCs were incubated with anti-CD16/CD32 antibody for blockade of Fc receptors before staining. Dead cells were excluded using Zombie NIR™ Fixable Viability Kit (4,423,105, Biolegend) and cell surfaces were stained using anti-CD45, CD11b, F4/80, and CD11c antibodies. Samples were detecteded by FACSCantoII (BD Biosciences, USA). FlowJo software (v10.10.0, Tree Star, USA) was used for data analysis. The antibody information was shown in Supplementary Table 4.

### In vitro stimulation of BMDMs/BMDCs

BMDM of WT and *Aim2*^*−/−*^ mice were infected with live *C. albicans* (MOI = 5) for 30 min to detect the activation of Akt signaling pathway.

To detect apoptosis, live *C. albicans* (MOI = 5) was used to infect cells at 37 °C with 5% CO_2_. Following an 8-h infection period, cells were lysed to determine the level of cleaved-Caspase3/7 protein. Cell death was determined using diluted Sytox Green dead cell nucleic acid dye, and observation via fluorescence microscopy (Leica DMi8).

For DNA transfection, each reaction consisted of 2.5 μg of poly (dA: dT) (InvivoGen; tlrl-patn) resuspended in PBS and mixed with 100 μl of LyoVec™ reaction buffer (InvivoGen). After 15 min, DNA complexes were added to cells and incubated for 30 min and 8 h, respectively. *C. albicans* DNA was extracted using the Fungal Genome DNA Extraction Kit according to the manufacturer’s instructions (Solarbio, China). DNA transfection was performed as described above.

### Immunofluorescence microscopy

The slides of BMDMs were blocked with 3% BSA and then incubated with Sytox nucleic acid dye (488 nm) and anti-mouse F4/80-PE (561 nm) antibody at appropriate dilution. Then, the slides were rinsed with cold normal saline and the cells were mounted with Antifade Reagent with DAPI (Solarbio, China). Images were acquired using a Leica confocal microscope (STELLARIS 5, Leica, Germany).

### Clodronate liposme treatment

To determine the effect of macrophage on *C. albicans* infection, clodronate liposome (ClodroLip, Yeasen Biotech, China) was administered intravenously to mice at a dose of 1 mg/20 g body weight (BW) for two times starting 2 days before infection to delete macrophage. PBS—encapsulated liposomes were administered in the same manner to the control group. After that, the mice were infected with *C. albicans.*

### Macrophage cytotoxicity and phygocytosis assay

For the phagocytosis assay, live *C. albicans* (MOI = 10) were co-incubated with WT and *Aim2*^*−/−*^ mice BMDM for one hour, after which amphotericin B was introduced to eliminate extracellular fungi. One hour later, the cells were lysed using 1% Triton and diluted in Sabouraud plates. Following incubation at 30 °C for 24 h, the colonies were enumerated to indicate phagocytic capacity. The killing assay was conducted as previously described with some modifications [11]. Following incubation, cells were disrupted using 1% Triton and plated for CFU counting to determine killing ability. The percentage of killing was calculated using the following formulas:　Survival % = dilution factor * 24 survival CFU / 2 h phagocytosis * 100; Killing % = 100%—Survival %.

### AKT inhibitor treatment

In order to evaluate the potential therapeutic efficacy of Akt inhibition, *Aim2*^*−/−*^ mice were initially infected with *C. albicans* intravenously at a concentration of 2 × 10^5^ CFU. Subsequently, the mice were treated with API-2 (#2151, Tocris, USA), a selective Akt inhibitor, at a dosage of 1 mg/kg/d via intraperitoneal injection for a duration of 2 days.

### Statistical analysis

The data were analyzed using GraphPad Prism software 9.4.1 (GraphPad Software, La Jolla, CA, USA). The data with a normal distribution were shown as means ± SEM. The unpaired two-tailed Student’s t-test was used for comparison between two groups, while one-way ANOVA was used for comparison among multiple groups in a univariate design, and two-way ANOVA with repeated measures wass used for comparison among multiple groups with two factor design, considering the interaction between two factors. The data without normal distribution were shown as median and analyzed by non parametric test (Mann–Whitney test). Survival curves were evaluated using the log-rank test. *P* < 0.05 was considered statistically significant.

## Results

### AIM2 enhances systemic *C. albicans* infection and inflammatory responses

**Fig. 1:**
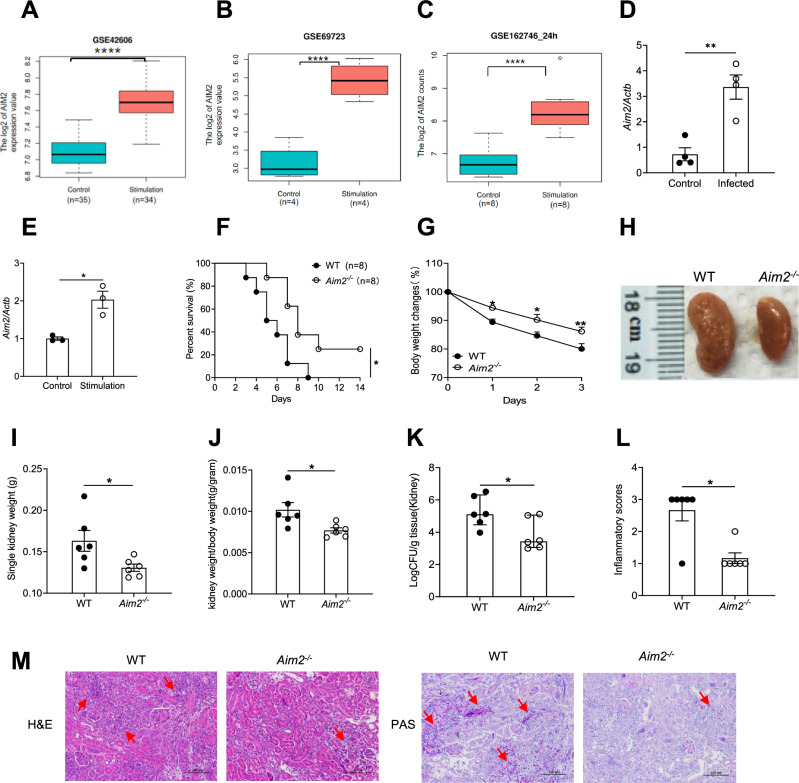
The *Aim2*^*−/−*^ mice are resistant to *C. albicans* infection. **A-C** The gene expression of AIM2 were determined by RNA-seq. **A** The peripheral blood mononuclear cells (PBMCs) from healthy volunteers were treated with either RPMI medium (Control, n = 35) or *C. albicans* (Stimulation, n = 34) for 24 h. Data were sourced from GSE42606. **B** Monocyte-derived dendritic cells (DCs) of 4 human donors were co-cultured with either RPMI medium (Control) or *C. albicans* (MOI = 1, Stimulation) for 6 h. Data were sourced from GSE69723. **C** Human PBMCs from 8 healthy volunteers were treated with either RPMI medium or *C. albicans* for 24 h. Data were sourced from GSE162746. **D-E** The gene expression levels of Aim2 were determined by RT-qPCR. **D** WT mice were either treated with PBS (Control, n = 4) or *C. albicans* (Infected, n = 4) and the kidney tissues were collected for Aim2 expression analysis. **E** BMDMs were treated either PBS (Control, n = 3) or *C. albicans* (Stimulation, n = 3) for 3 h and cells were collected for Aim2 expression analysis. **F-M** WT and *Aim2*^*−/−*^ mice were subjected to intravenous infection with *C. albicans* (2 × 10^5^ CFU) and subsequently monitored over time. **F** Kaplan–Meier survival plots were generated for both WT (n = 8) and *Aim2*^*−/−*^ (n = 8) mice. At 3 days post-infection, mice (n = 6) were sacrificed and various parameters were evaluated, including changes in body weight (**G**), gross kidney morphology (**H**), kidney weights (**I**), kidney weights relative to body weight (**J**), kidney CFU (**K**), and histological analysis of kidneys using H&E and PAS staining (**M**), red arrows indicate infiltration of inflammatory cells in H&E staining and *C. albicans* in PAS staining; Histological lesion scores were also determined for each group (**L**). The results are representative of two (**L, M**) or three (**D-K**) independent experiments. Data are shown as means ± SEM. **F** Log-rank test, **G** two-way ANOVA with Holm-Sidak’s multiple comparisons test, **A-E, I-J** Unpaired two-tailed Student’s *t*-test and **K, L** Mann–Whitney test. *P < 0.05, **P < 0.01 and ****P < 0.0001

To understand the role of AIM2 in defense against systemic *candidiasis*, we sourced bulk RNA-seq data from peripheral blood mononuclear cells (PBMCs) or monocyte-derived dendritic cells of healthy volunteers challenged ex vivo with *C. albicans* (GSE69723, GSE42606, GSE162746) [38–40]. Compared to those of unstimulated controls, transcription of *AIM2* was significantly elevated after *C. albicans* stimulation (Fig. 1A–C). To determine the function of AIM2 gene, we next extracted the raw data from these three databases, then calculated Gene ontology (GO) enrichment scores using the Database for Annotation, Visualization and Integrated Discovery (DAVID). The biological functions of AIM2 relevant genes were found to be focused on defense response to virus, inflammatory response, innate immune response and apoptotic process after *C. albicans* infection. (Supplementary Table 5).

To validate this result in vivo and vitro of murine model, WT mice were infected with *C. albicans* (2 × 10^5^ CFU) and kidneys were collected at 3 days post-infection. For *vitro* experiment, BMDMs from WT mice were challenged with *C. albicans* for 3 h*.* We found significant induction of the same *Aim2* gene after *C. albicans* infection (Fig. 1D–E). Collectively, these data show that activation of *Aim2* transcription both in human and mice are one of the biomarkers of *candidemia*.

In order to investigate the possible involvement of AIM2 in host defense against systemic fungal infection, WT and *Aim2*^*−/−*^ mice were chosen for systemic *C. albicans* infection. The results showed that *Aim2*^*−/−*^ mice exhibited resistance to *C. albicans* infection in comparison to WT mice, as evidenced by enhanced survival rates (Fig. 1F), mitigation of infection-induced body weight loss (Fig. 1G), and smaller single kidney sizes or kidney/body weight rations (Fig. 1H–J). Moreover, *Aim2*^*−/−*^ mice demonstrated a significant reduction in fungal burdens in the kidneys (Fig. 1K). The histopathological examination of kidneys post-infection confirmed these outcomes, with *Aim2*^*−/−*^ mice exhibiting reduced renal damage (Fig. 1L–M) as observed through H&E staining, and decreased *C. albicans* quantities as shown by PAS staining (Fig. 1M). These results show that AIM2 enhances systemic *C. albicans* infections.

**Fig. 2:**
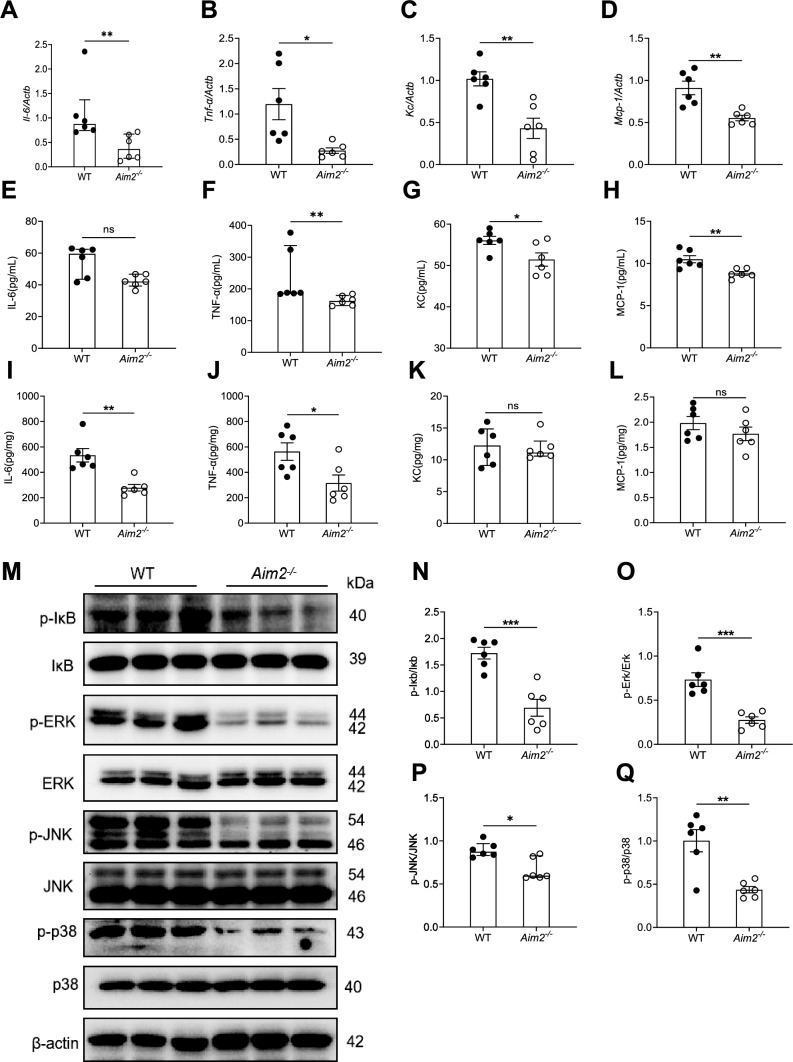
Attenuated inflammation in *Aim2*^*−/−*^ mice after *C. albicans* infection. WT and *Aim2*^*−/−*^ mice were subjected to *C. albicans* infection and the kidneys were harvested at 3 days post-infection. The expression levels of proinflammatory cytokines were assessed. **A-D** *Il6*, *Tnf-a*, *Kc*, and *Mcp-1* gene expression in the kidneys were determined by RT-qPCR and **E-L** IL-6, TNF-ɑ, KC, MCP-1 protein expression in sera and kidneys were determined by ELISA. **M** The protein levels of p-IκB/IκB, p-ERK/ERK, p-JNK/JNK, p-p38/p-38, and β-actin were determined by Western-blot and the representative images were shown. Each lane represents samples from different mice. **N-Q** The densitometric analysis of Western-blot results in (**M**). **A-L, N-Q** n = 6 mice/group. The results are representative of three independent experiments. Data are shown as means ± SEM. **B-D, G-J, L, N–O, Q** Unpaired two-tailed Student’s *t*-test. **A, E–F, K, P** Mann–Whitney test. *P < 0.05, **P < 0.01, ***P < 0.001, and ns, not statistically significant

In accordance with the resistance observed in *Aim2*^*−/−*^ mice towards *C. albicans* infection, AIM2 deficiency results in a reduction of inflammation levels subsequent to infection. Specifically, the expression levels of pro-inflammatory cytokine genes, such as *Il6*, *Tnf-ɑ*, *Kc*, and *Mcp-1*, were determined through RT-qPCR and found to be significantly downregulated in the kidneys of *Aim2*^*−/−*^ mice compared to controls (Fig. 2A–D). Additionally, the levels of pro-inflammatory cytokines, including IL-6, TNF-ɑ, KC, and MCP-1, were measured through ELISA and were found to be significantly lower in the sera and kidneys of *Aim2*^*−/−*^ mice compared to controls, with the exception of TNF-ɑ in sera and KC and MCP-1 in kidneys (Fig. 2E–L). The Western-blot analysis conducted on inflammatory pathways, namely NF-κB and MAPKs, revealed that *Aim2*^*−/−*^ mice kidneys exhibited significantly lower levels of activation of p-IκB/IκB, p-ERK/ERK, p-JNK/JNK, and p-p38/p38 in comparison to their WT counterparts following infection (Fig. 2M–Q).

### AIM2 enhances systemic *C. albicans* infection independently of inflammasome or Type I interferon (IFN-I) production

**Fig. 3:**
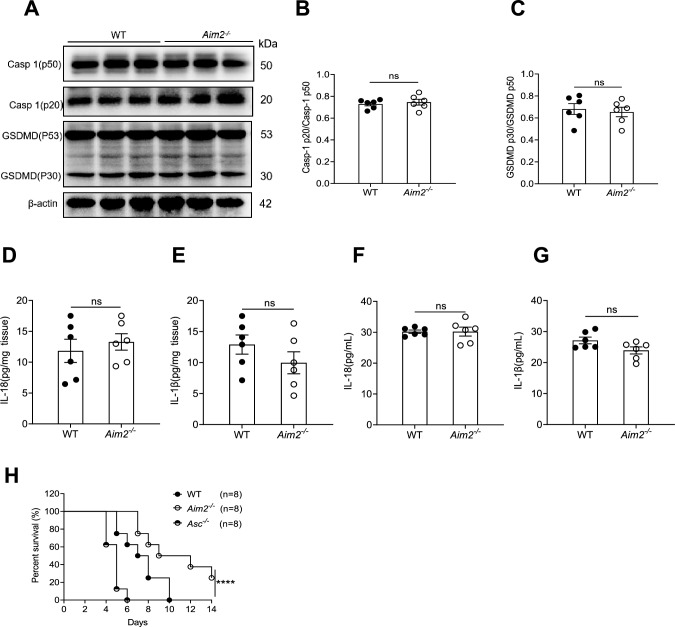
The resistance of *Aim2*^*−/−*^ mice to *C. albicans* infection is independently of inflammasome activation. WT and *Aim2*^*−/−*^ mice were subjected to *C. albicans* infection. The kidneys and sera were harvested at 3 days post-infection. **A** The protein levels of Caspase-1 (Casp1) and Gasdermin-D (GSDMD) were analyzed using Western-blot. Each lane represents samples from different mice. **B-C** The densitometric analysis of Western-blot results in (**A**). **D-G** The protein expression levels of IL-18 and IL-1β in the kidneys (**D-E**) and sera (**F-G**) were quantified using ELISA. **H** WT, *Aim2*^*−/−*^, and *Asc*^*−/−*^ mice were infected with *C. albicans* and their survival rates were monitored over time. **B-G** n = 6 mice/group, and (**H**) n = 8 mice/group. The results are representative of two (**H**) or three independent experiments (**A-G**). Data are shown as means ± SEM. **B-G** Unpaired two-tailed Student’s *t*-test and (**H**) Log-rank test. ****P < 0.0001, and ns, not statistically significant

In order to investigate whether the resistance of *Aim2*^*−/−*^ mice to *C. albicans* infection is linked to AIM2 inflammation, we examined the inflammasome activation levels in the kidneys of WT and *Aim2*^*−/−*^ mice after infection. The findings indicate that the levels of activated Caspase-1 in the kidneys, as determined by Western-blot analysis, were similar between WT and *Aim2*^*−/−*^ mice (Fig. 3A–B). Gasdermin D (GSDMD) has been identified as a crucial factor responsible for the inflammatory form of pyroptotic cell death, which is cleaved by Caspase-1[41]. Our findings indicate that there is no discernible difference in the expression of GSDMD between WT and *Aim2*^*−/−*^ mice (Fig. 3A, C). Additionally, we observed similar levels of cleaved IL-1β and IL-18 proteins in the sera and kidneys of both WT and *Aim2*^*−/−*^ mice, as determined by ELISA (Fig. 3D–G). To further investigate the role of inflammasomes in *C. albicans* infection, we utilized inflammasome-defective mice (*Asc*^*−/−*^ mice) and compared the mortality rates of WT, *Aim2*^*−/−*^, and *Asc*^*−/−*^ mice*.* Consistently, *Asc*^*−/−*^ mice succumbed to *C. albicans* infection within 6 days, while WT mice survived for up to 10 days. Notably, 50% of *Aim2*^*−/−*^ mice remained alive at day 10 post infection (Fig. 3H). These findings suggest that AIM2 plays an inflammasome-independent role in *C. albicans* infection, and that its response differs markedly from that of *Asc*^−/−^ mice.

IFN-I is known to play a critical role in host defense against *C. albicans* infection, and may serve as a hallmark of protective innate immunity or contribute to fatal immunopathology during *Candida* infections [42]. In order to assess the potential impact of IFN-I on the susceptibility of *Aim2*^*−/−*^ mice to *C. albicans* infection, we conducted an investigation into the gene and protein expression of IFN-I in the kidneys of both WT and *Aim2*^*−/−*^ mice following infection. Our findings indicate that there were no significant differences observed between the two groups (Figure S2A–D). Furthermore, we also evaluated the activation levels of kinase TBK1 and transcription factors IRF3 and IRF7, which are known to regulate IFN-I production, and found that they were comparable in the kidneys of both WT and *Aim2*^*−/−*^ mice (Figure S2E–H). These results suggest that the resistance of *Aim2*^*−/−*^ mice to *C. albicans* infection may not be attributed to IFN-I production.

### AIM2 promotes cell apoptosis in kidneys after *C. albicans* infection

Programmed cell death is a crucial component of the host’s innate immune defense against pathogens, and certain physiological processes nacessitate cell death to maintain functionality. Apoptosis, a form of programmed cell death, plays a vital role in immune system homeostasis [43]. However, the failure of apoptosis or excessive activation of apoptosis can lead to various diseases [44]. Studies have demonstrated that *C. albicans* infection induces cell apoptosis [45], and AIM2 has been found to activate caspase-8-mediated apoptosis [46].

**Fig. 4:**
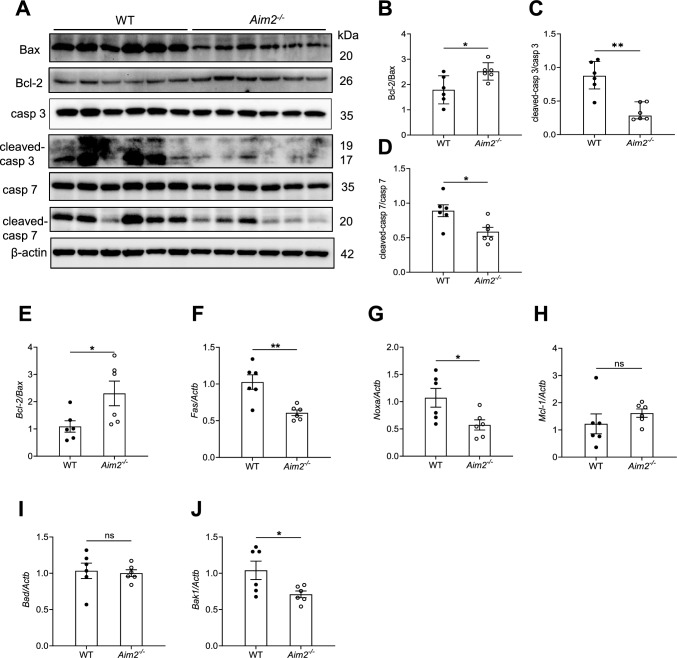
AIM2 induces cell apoptosis in kidneys after *C. albicans* infection. WT and *Aim2*^*−/−*^ mice were subjected to *C. albicans* infection*,* and the kidneys were harvested at 3 days post-infection. **A** The protein levels of Bcl2/Bax, cleaved-caspase3/caspase 3, cleaved-caspase 7/caspase 7 and β-actin in the kidneys were determined using Western-blot. Each lane represents samples from different mice. **B-D** The densitometric analysis of Western-blot results in (**A**). **E-J** The gene expression levels of apoptosis-related genes in the kidneys were determined using RT-qPCR. **E-J** n = 6 mice/group. The results are representative of three independent experiments. Data are shown as means ± SEM. **B, D-J** Unpaired two-tailed Student’s *t*-test. **C** Mann–Whitney test. * *P* < 0.05, **P < 0.01 and ns, not statistically significant

Therefore, we proceeded to investigate apoptosis in the kidneys of both WT and *Aim2*^*−/−*^ mice following *C. albicans* infection*.* Bax is one of pro-apoptotic molecules and Bcl2 is one of the anti-apoptotic molecules, therefore the ratio of Bcl2/Bax represents the ability of anti-apoptosis [47].The present study reveals that the Bcl2/Bax ratio was found to be higher in the kidneys of *Aim2*^*−/−*^ mice as compared to the control group following infection. It indicates that *Aim2*^*−/−*^ mice displayed stronger anti-apoptosis ability compared to WT mice **(**Fig. 4A). Furthermore, the activation levels of apoptosis executioners Caspase 3 (p17/19) and Caspase 7 (p20) were significantly reduced in the kidneys of *Aim2*^*−/−*^ mice (Fig. 4A–D).

Additionally, the mRNA expression levels of apoptosis-related genes, including *Fas, Noxa,* and *Bak1,* but not *Mcl-1* and *Bad* were significantly decreased in the kidneys of *Aim2*^*−/−*^ mice when compared to those of WT mice (Fig. 4E–J). These observations suggest that AIM2 plays a crucial role in promoting cell apoptosis in the kidneys following *C. albicans* infection.

To rule out whether all of the above differential effect can occur in steady state, the expression of inflammatory molecules, inflammasome, type I interferon and apoptosis proteins were detected in kidneys from uninfected WT and *Aim2*^*−/−*^ mice by Western-blot. No significant difference of those signaling pathways was observed in kidneys between WT and *Aim2*^*−/−*^ mice (Figure S3A-D).

### AIM2 in macrophages, but not in dendritic cells, enhances systemic *C. albicans* infection

**Fig. 5:**
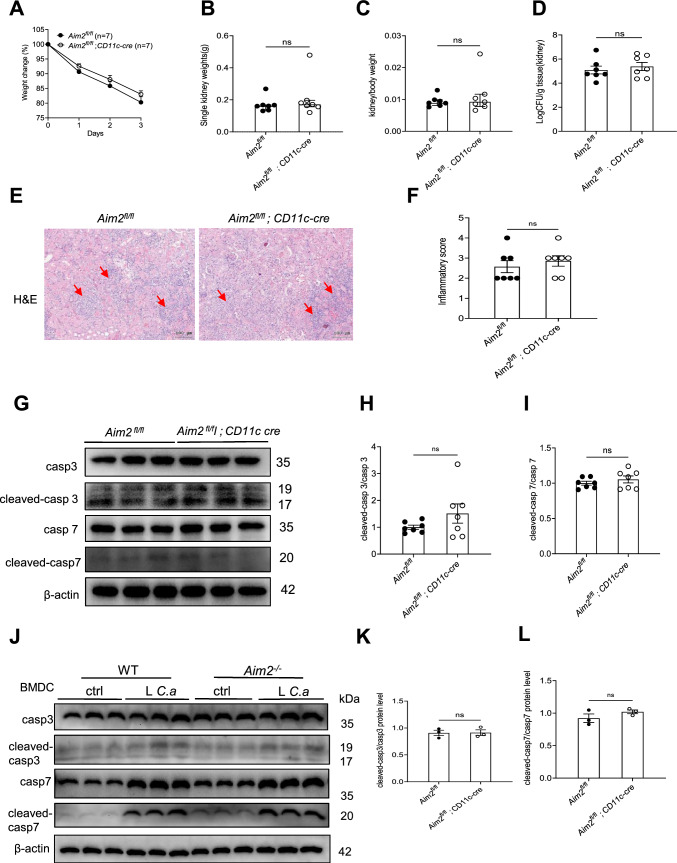
AIM2 in dendritic cells does not affect the host defense aganist *C. albicans* infection. *Aim2*^*fl/fl*^ and *Aim2*^*fl/fl*^*; CD11c-cre* mice were subjected to *C. albicans* infection and subsequently sacrificed at 3 days post-infection. The following parameters were evaluated: **A** body weight changes, **B** kidney weights, **C** kidney weights relative to body weights, **D** CFU in kidneys, **E** H&E-stained kidney sections and red arrows indicate infiltration of inflammatory cells, **F** histological lesion scores. **G** The protein levels of cleaved-caspase 3/caspase 3, cleaved-caspase 7/caspase 7 and β-actin in the kidneys were determined using Western-blot. Each lane represents samples from different mice. **H-I** The densitometric analysis of Western-blot results in (G). BMDCs from WT and *Aim2*^*−/−*^ mice were either uninfected or infected with *C. albicans* for 8 h at MOI 5. **J** The protein levels of cleaved-caspase 3/caspase 3, cleaved caspase 7/caspase 7 and β-actin in BMDCs without or with the stimulation of *C. albicans*. **K-L** The densitometric analysis of Western-blot in (**J**). **A**–**D**, **F**, **H**–**I** n = 7 mice/group. The results are representative of two independent experiments. The data are shown as means ± SEM. **A** Two-way ANOVA with Holm-Sidak’s multiple comparisons test, **D, H-I, K-L** Unpaired two-tailed Student’s *t*-test and **B-C, F** Mann–Whitney test. ns, not statistically significant

The gene AIM2 is highly expressed in innate immune cells, specifically macrophages and dendritic cells [48]. Consistently, our results showed that AIM2 was expressed both in dendritic cells and macrophages (Figure S4A). Given its known role in enhancing systemic *C. albicans* infection, we sought to investigate the specific contribution of AIM2 in macrophages and dendritic cells to this process. To this end, we generated mice with AIM2 deficiency in either macrophages (*Aim2*^*fl/fl*^*; Lyz-cre* mice) or dendritic cells (*Aim2*^*fl/fl*^*; CD11c-cre* mice), as well as their respective controls (*Aim2*^*fl/fl*^ mice). Upon infection with *C. albicans*, *Aim2*^*fl/fl*^*; CD11c-cre* mice exhibited comparable responses to A*im2*^*fl/fl*^ mice, as evidenced by similar changes in body weight, kidney weights, kidney CFU, and histological lesions (Fig. 5A–F). We further examined the extent of apoptosis damage attributed to the AIM2 expression on CD11c^+^ DC by Western-blot analysis. As expected, apoptosis in kidneys from control and *Aim2*^*fl/fl*^*; CD11c-cre* mice were similarly increased after *C. albican* infection (Fig. 5G–I). To confirm the effect of AIM2 on dendritic cells, we performed the experiments using BMDCs and found that *C. albicans* infection induced similar levels of cleaved-caspase-3/7 in both WT and *Aim2*^−/−^ BMDCs (Fig. 5J–L).

**Fig. 6:**
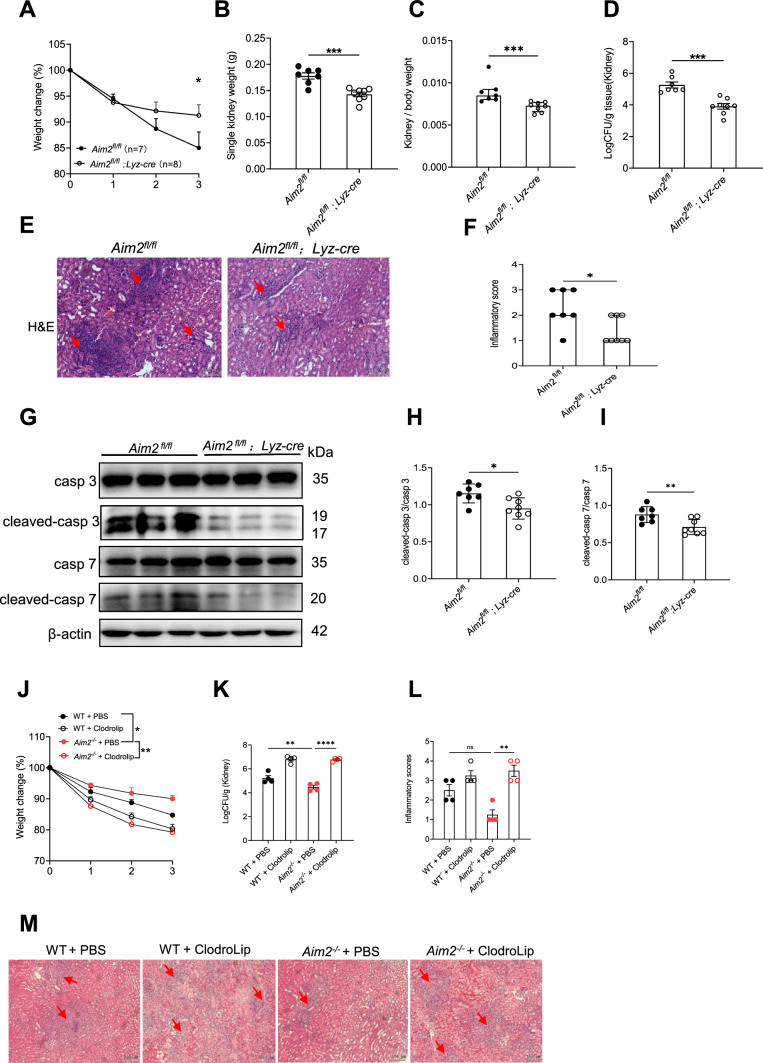
AIM2 in monocytes/macrophages enhances *C. albicans* infection. *Aim2*^*fl/fl*^ and *Aim2*^*fl/fl*^*; Lyz-cre* mice were subjected to *C. albicans* infection and subsequently sacrificed at 3 days post-infection. The following parameters were assessed: **A** body weight changes, **B** kidney weights, and **C** kidney weights relative to body weights. **D** CFU in kidneys, **E** H&E-stained kidney sections and red arrows indicate infiltration of inflammatory cells, **F** histological lesion scores. **G** The protein levels of cleaved-caspase3/caspase 3 and cleaved-caspase 7/caspase 7 and β-actin in the kidneys were determined using Western-blot. Each lane represents samples from different mice. **H, I** The densitometric analysis of Western-blot in (G). The results are representative of two independent experiments. WT and *Aim2*^*−/−*^ mice were injected twice with PBS or ClodroLip before *C. albicans* infection and mice were sacrificed at 3 days post-infection. **J** body weight changes, **K** CFU in kidneys, **M** H&E-stained kidney sections and red arrows indicate infiltration of inflammatory cells, **L** histological lesion scores. Data are shown as means ± SEM. **A, J** Two-way ANOVA with Holm-Sidak’s multiple comparisons test, **B, D, H-I** unpaired two-tailed Student’s *t*-test, **C, F** Mann–Whitney test and **K** one-way ANOVA with Fisher’s LSD test*.* **L** non parametric test*.* *P < 0.05, **P < 0.01, ***P < 0.001, ****P < 0.0001 ns, not statistically significant

In contrast to *Aim2*^*fl/fl*^*; CD11c-cre* mice, *Aim2*^*fl/fl*^*; Lyz-cre* mice exhibited resistance to *C. albicans* infection, as evidenced by reduced body weight loss, kidney weight, kidney CFU, and histological lesions compared to A*im2*^*fl/fl*^ mice following infection (Fig. 6A–F). Consistent with *Aim2*^*−/−*^ mice, apoptosis damage in kidneys from *Aim2*^*fl/fl*^*; Lyz-cre* mice were markedly decreased (Fig. 6G–I). To reconfirm the role of monocytes/macrophages in *C. albicans* infection, Clodronate liposome was used to depletes macrophages. It was observed that treatment with ClodroLip eliminated the difference in susceptibility to *C. albicans* infection between WT mice and *Aim2*^*−/−*^ mice, as evidenced by similar changes in body weight, kidney CFU, and pathological damage (Fig. 6J–M). Together, these data strongly support a role for AIM2 expression in monocytes/macrophages, but not dendritic cells, enhances susceptibility to *C. albicans* infection.

The innate host defense against fungal pathogens is largely mediated by phagocytosis and killing by macrophages [49]. In order to directly examine the impact of Aim2 deficiency on macrophage functions, we conducted phagocytosis and killing assays through co-culturing BMDMs and *C. albicans*. It is noteworthy that no significant difference in phagocytosis and fungicidal activity was observed between WT and *Aim2*^*−/−*^ BMDMs (Figure S5A–B).

**Fig. 7:**
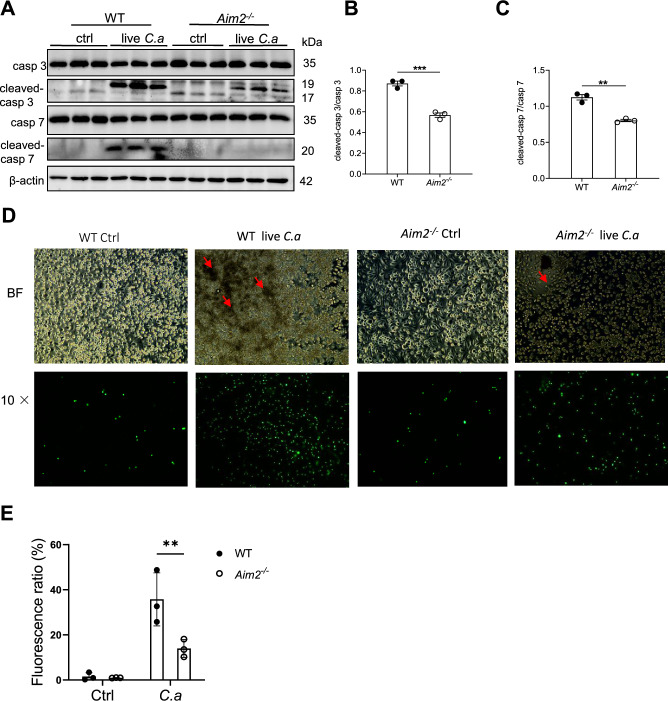
AIM2 promotes apoptosis of macrophages after *C. albicans* infection. BMDMs from WT and *Aim2*^*−/−*^ mice were either uninfected or infected with live *C. albicans* for 8 h at MOI 5. **A** The protein levels of cleaved-caspase 3/caspase 3, and cleaved caspase-7/caspase 7 in macrophages were determined by Western-blot. **B, C** Densitometric analysis of the Western-blot results in (**A**). **D** Cell death in BMDMs after *C. albicans* infection, measured by SYTOX Green uptake assay. red arrows indicate the hyphae, green indicates dead cells. **E** The densitometric analysis of (D). The results are representative of three independent experiments. Data are shown as means ± SEM. **B-C** Unpaired two-tailed Student’s *t*-test*.* **E** Two-way ANOVA with Holm-Sidak’s multiple comparisons test. **P < 0.01 and ***P < 0.001

In order to gain a more comprehensive understanding of the underlying cellular mechanisms of the observed protective effect, we evaluated apoptosis in BMDMs from both WT and *Aim2*^*−/−*^ mice. Specifically, the BMDMs were infected with *C. albicans* (MOI = 5) for a duration of 8 h. Our findings are consistent with in vivo results, as we have observed a reduction in the activation of apoptosis-associated molecules, Caspase 3 and Caspase 7, in A*im2*^*−/−*^ BMDMs compared to WT BMDMs following *C. albicans* stimulation (Fig. 7A–C). In addition, *C. albicans* DNA and Poly (dA: dT) were used to transfect BMDMs. The results showed that AIM2 was activated by DNA from *C. albicans* and apoptosis activation was reduced in *Aim2*^*−/−*^ BMDMs in compaison with controls after the treatmetn of *C. albicans* DNA or Poly (dA: dT) (Fig. S6A). Subsequent analysis utilizing fluorescent microscopy revealed a decrease in cell death in A*im2*^*−/−*^ BMDMs relative to controls post infection (Fig. 7D–E). We further demonstrated that the apoptosis occured in macrophage, as Sytox (green fluorescence) and F4/80 (red fluorescence) were co-localized under confocal microscopes (Figure S7A). These results suggest that AIM2 promotes *C. albicans* infection by facilitating apoptosis, rather than phagocytosis or macrophage killing.

### Akt mediates AIM2-induced cell apoptosis and resistance against *C. albicans* infection

**Fig. 8:**
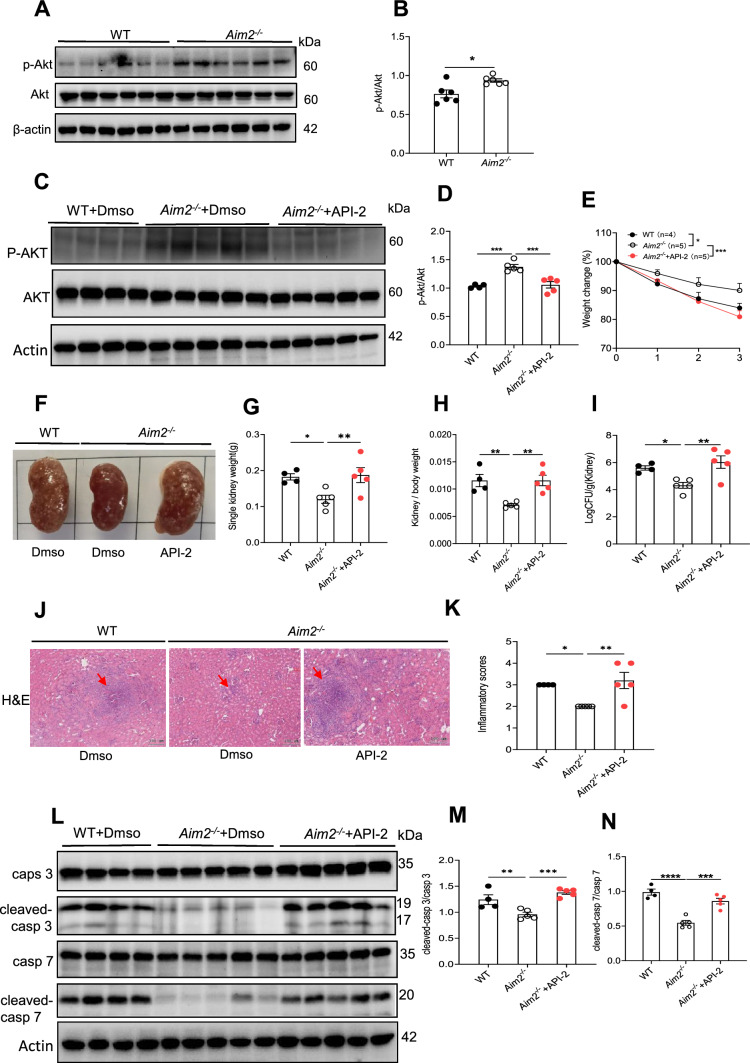
AIM2 promotes cell apoptosis via inhibiting Akt activation. A-B) WT and *Aim2*^*−/−*^ mice were subjected to *C. albicans* infection*,* and their kidneys were collected at 3 days post-infection. **A** The protein levels of p-AKT/AKT in the kidneys were determined using Western-blot analysis. **B** Densitometric analysis of the Western-blot in (**A**). **C-N** WT and *Aim2*^*−/−*^ mice were infected with *C. albicans*, and *Aim2*.^*−/−*^ mice were injected with either DMSO or AKT inhibitor API-2. Mice were sacrificed at 3 days after infection. **C** The protein levels of p-AKT/AKT determined by Western-blot. **D** Densitometric analysis of Western-blot results in (**C**). **E** the body weight changes, and **F** gross pictures of the kidneys were recorded. **G** Kidney weights. **H** Kidney weights /Body weight. **I** Kidney CFU. **J** H&E-stained kidney sections and red arrows indicate infiltration of inflammatory cells. **K** Histological lesion scores. **L** The protein levels of cleaved-Caspase 3/Caspase 3, and cleaved Caspase-7/Caspase 7 determined by Western-blot. **M, N** Densitometric analysis of Western-blot results. Each lane represents samples from different mice. The results are representative of two (**C-N**) or three (**A-B**) independent experiments. Data are shown as means ± SEM. **B** Unpaired two-tailed Student’s *t*-test, (**D, G-I, and M–N**) One-way ANOVA with Fisher’s LSD test and (**E**) Two-way ANOVA with Holm-Sidak’s multiple comparisons test. **K** one-way ANOVA with Fisher’s LSD test*.* *P < 0.05, **P < 0.01, ***P < 0.001 and ****P < 0.0001. (A, C, L)

AIM2 can regulate tumor development or host defense by inhibiting the Akt signaling pathway independent of inflammasome [31–33, 50], while Akt phosphorylation has been shown to attenuate apoptosis [51–54]. The present study aimed to investigate the potential role of Akt activation, regulated by AIM2, in mediating cell apoptosis and resistance against *C. albicans* infection. The phosphorylation level of Akt was suppressed in kidneys of WT mice following *C. albicans* infection compared to uninfected controls (Figure S8A-B). The Further data indicated that *Aim2*^*−/−*^ mice exhibited a higher level of Akt phosphorylation in kidneys compared to WT mice (Fig. 8A–B). Consistent with *Aim2*^*−/−*^ mice, p-Akt from *Aim2*^*fl/fl*^*; Lyz-cre* mice rather than *Aim2*^*fl/fl*^*; CD11c-cre* mice was significantly increased (Figure S8C-F). We also used *C. albicans* to stimulate BMDM and observed the similar results (Figure S8G).

To further explore this relationship, *Aim2*^*−/−*^ mice were administered a highly specific Akt inhibitor (API-2) via intraperitoneal injection for a period of 2 days following *C. albicans* infection. Notably, the level of phosphorylated Akt in kidneys was significantly suppressed in API-2-treated *Aim2*^*−/−*^ mice (Fig. 8C–D). Furthermore, it was observed that API-2-treated *Aim2*^*−/−*^ mice experienced greater body weight loss, kidney weight, kidney CFU, and histological lesions compared to DMSO-treated *Aim2*^*−/−*^ mice after *C. albicans* infection (Fig. 8E–K). The present study consistently demonstrates that API-2 treatment in *Aim2*^*−/−*^ mice resulted in higher levels of active caspase-3/7 protein expression in the kidneys compared to DMSO-treated *Aim2*^*−/−*^ control mice following infection with *C. albicans* (Fig. 8L–N). These findings suggest that AIM2 plays a crucial role in inducing macrophage apoptosis and promoting host resistance against *C. albicans* infection by inhibiting Akt activation.

## Discussion

Intracellular PRRs have been shown to be critical in the host defense against *C. albicans* infection, with NLRP3 forming the inflammasome and inducing the release of IL-1β and IL-18 to eliminate *C. albicans* [13, 55]. Nonetheless, a separate investigation demonstrated that inflammasome-mediated pyroptosis could facilitate *C. albicans* infection [56]. The intracellular receptor NLRP10 induced the release of cytokines IFN-γ and IL-17 to combat *C. albicans* infection [57]. NLRC4 mitigated mucosal *C. albicans* infection by diminishing inflammatory cell recruitment and the generation of antimicrobial peptides [14]. The identification of novel roles of intracellular PRRs will enhance our understanding on the innate immune mechanism underlying host defense against *C. albicans* infection.

As a cytoplasmic DNA sensor, AIM2 has been identified as a key player in the recognition of dsDNA from microorganisms, thereby contributing to host resistance against various pathogenic microorganisms, such as *Francisella tularensis*, *Streptococcus*, *Plasmodium*, Epstein-Barr virus, and influenza virus [46, 58]. AIM2 also detects self-derived DNA with noted roles in the immune response to tumors, radiation-induced tissue damage, and the DNA-damage response in mouse models of neurodevelopment, polyarthritis, and atherosclerosis [59].

Compared to bacterial and viral infection, the role of the AIM2 in response to fungal infection is less clear. A study shows that AIM2 and NLRP3 cooperatively assembled inflammasome to control the *Aspergillus fumigatus* infection and AIM2 inflammasome itself had no significant effect on host resistance to *A. fumigatus* infection [34]. IRGB10 induced by *A. fumigatus* infection targeted *Aspergillus* hypha for the release of fungal ligands, which subsequently caused the activation of both AIM2 and NLRP3 inflammasome, thus promoting host resistance against *A. fumigatus* infection [60]. Moreover, AIM2, pyrin, and ZBP1 form a PANoptosome complex to sense *A. fumigatus* [61]. These studies suggest that AIM2 suppresses fungal infection by inflammasome or PANoptosome-dependent manner. Our previous studies and others have shown that nucleic acid-sensing PRRs such as MDA5 [62] and STING [63] enhanced *C. albicans* infection. In addition, it has also been reported that AIM2 regulate the development of colorectal cancer [31, 32] and host defense against *Salmonella* infection [33] in inflammasome-independent manner. Consistently, we found that clinical data from GEO database displays elevated expression levels of AIM2 in PBMCs or dendritic cells stimulated with *C. albicans*. However, the physiological role of AIM2 in host defense against *C. albicans* infection remains elusive.

In this study, we identify a novel function of AIM2 in macrophages during *C. albicans* infection. Surprisingly, our findings reveal that, in contrast to its protective role in other types of infection, nucleic acid-sensing PRR AIM2 actually enhances *C. albicans* infection. The spectrum of disease invasive *C. albicans* infection ranges from minimally symptomatic *candidaemia* to fulminant sepsis with an associated mortality exceeding 70% [64]. The inflammatory infiltration of the kidney in *Aim2*^*−/−*^ mice was markedly reduced. The reduced level of inflammation subsequently reduces the severity of sepsis in early infection, which may explain the resistance to *C. albicans* infection in *Aim2*^*−/−*^ mice.

We investigated the inflammasome activity in the kidneys of WT and *Aim2*^*−/−*^ mice following *C. albicans* infection, given the well-known propensity of AIM2 to form inflammasomes in conjunction with ASC and Caspase-1. Our findings suggest that the resistance of *Aim2*^*−/−*^ mice to *C. albicans* infection may not be linked to inflammasome activity. It was reported that *C. albicans* exclusively activates NLRP3 inflammasome, rather than AIM2 inflammasome [34]. Similarly, our results showed that AIM2 deficiency did not result in attenuated inflammasome activation, possibly due to the ability of *C. albicans* to activate other inflammasome signaling pathways via the activation of other PRRs, such as NLRP3.

It is worth noting that AIM2 has been reported to be an interferon-inducible protein [65]. It has been reported that cGAS/IFI16- STING—type I IFN signaling can promote AIM2 upregulation [66]. Following this, an analysis was conducted on the activation levels of the interferon signaling pathway, which revealed that both WT and *Aim2*^*−/−*^ mice exhibited a comparable expression level of type I interferons subsequent to *C. albicans* infection.

It is widely acknowledged that macrophages exert regulatory effects in various pathological processes [67]. Our research demonstrated that AIM2 in macrophages, but not in dendritic cells, enhances *C. albicans* infection. Furthermore, through co-culturing of *C. albicans* and macrophages, we observed no significant difference in phagocytosis and fungicidal activity between WT and *Aim2*^*−/−*^ macrophages.

Physiologically, apoptosis serves to eliminate senescent, damaged, or mutated cells to maintain host homeostasis [43]. However, pathological apoptosis can contribute to the onset and progression of numerous diseases. Within the caspase cascade, caspase-3 is a pivotal pro-apoptotic protein that cleaves various substrates to amplify apoptosis signals and induce cell death [68]. Similarly, caspase-7 also facilitates cell death by delaying pore-driven lysis [69]. The findings of our study demonstrate that *C. albicans* can induce macrophage apoptosis both in vivo and in vitro, which has important implications for the pathogen’s ability to evade the host immune response. Our investigation also revealed that *Aim2*^*−/−*^ mice exhibit reduced expression of certain pro-apoptotic genes in the kidneys, as well as a higher Bcl2/Bax ratio, which is indicative of increased anti-apoptotic activity. Furthermore, the levels of cleaved-caspase3 and cleaved-caspase7 were significantly lower in *Aim2*^*−/−*^ mice compared to the control group. AIM2 is also implicated in a complicated cell death pathway, named PANoptosis, composing of simultaneous activation of pyroptosis, apoptosis, and necroptosis [70]. Research indicates that AIM2 form a complex with pyrin and ZBP1 to execute this form of inflammatory cell death [71]. Regrettably, we have not yet studied the other PANoptosis forms except apoptosis during *C. albicans* infection.

An increasing number of studies indicate that AIM2 has roles in immunity independent of the inflammasome response. In the murine AOM/DSS model of colorectal cancer, AIM2 was found to suppress AKT activation through interaction with DNA-dependent protein kinase to control tumor development [32] and suppress intestinal stem cell proliferation [31]. Other researches found that AIM2 negatively regulates the DNA-PK-AKT3 in microglia to control neuroinflammation [72] and enhances the stability of T regulatory cells via interacting with the RACK1-PP2A phosphatase complex to restrain AKT phosphorylation [50] in experimental autoimmune encephalomyelitis (EAE). Furthermore, AIM2 was shown to bind neutrophil extracellular traps, leading to DNase resistant nucleoprotein fibers that can serve as autoantigens in systemic lupus erythematosus (SLE) [73]. Another study [74] revealed that AIM2 regulates microglial activation during synaptic pruning in the dentate gyrus region via the complement pathway, leading to impaired synaptic plasticity and Pattern separation (PS) in aging mice. Those studies suggest that AIM2 has inflammasome-independent functions. Interestingly, we observed that Akt phosphorylation was higher in the kidneys of *Aim2*^*−/−*^ mice following *C. albicans* infection, suggesting a potential mechanism for the observed decrease in apoptosis. The reversal of cell apoptosis and resistance against *C. albicans* infection in *Aim2*^*−/−*^ mice was observed upon the administration of an Akt inhibitor. This led to the finding that AIM2 inhibits Akt signaling, thereby promoting cell apoptosis.

We provide a mechanistic analysis of AIM2-Akt-apoptosis axis in enhancing fungal infection. Furthermore, it is important to study whether AIM2 inhibits AKT phosphorylation to promote apoptosis by interacting with DNA-PK and/or other molecules. Future work is also needed to delineate the role of AIM2 in mucosal *Candidiasis*. Another study shows that nucleic acid sensor STING enhances *C. albicans* infection by suppressing CLR signaling pathway in dendritic cells [63]. It suggests that two nucleic acid sensors, AIM2 and STING, act through different mechanisms to enhance *C. albicans* infection. It can also be speculated that double-knockout (i.e., *Aim2*^*−/−*^* Sting*^*−/−*^) mice may show more resistance to *C. albicans* infection than single knockout mice. However, additional verification experiments are required.

Numerous studies have documented that AIM2 plays the protective roles against multiple diseases [29, 58, 75]. However, based on our current study, the overexpression of Aim2 may increase the risk of fungal infection in patients. Furthermore, our investigation also indicates that some small molecule such as AIM2 inhibitors can be applied to suppress invasive fungal infection. The research on the role of AIM2 in fungal infection is still limited to the experimental studies. The clinical researches can be conducted to explore the potential of manipulating AIM2 to control fungal infection in the future.

In summary, our investigation demonstrates that AIM2 enhances *C. albicans* infection by inducing macrophage apoptosis through AKT signaling, independent of inflammasome (Figure S9). The targeting of AIM2 or AKT may hold therapeutic implications for the treatment of systemic fungal infections.

### Supplementary Information

Below is the link to the electronic supplementary material.

Supplementary file1 (PDF 689 KB) Supplemental Figure 1. The validation of BMDMs/BMDCs. The single-cell suspensions of WT BMDMs and BMDCs were staining with antibodies and analyzed by flow cytometry. (A) Gating strategy of flow cytometry and the proportion of F4/80+ cells. (B) Gating strategy of flow cytometry and the proportion of CD11c+cells. Supplemental Figure 2. The resistance of Aim2-/- mice to C. albicans infection is not associated with the production of type I interferons. WT and Aim2-/- mice were subjected to C. albicans infection, and kidney, sera were collected at 3 days post-infection. The gene and protein expression levels of IFN-α and IFN-β were evaluated using RT-qPCR (A-B) and ELISA (C-D), respectively. Western-blot analysis was performed to determine the protein levels of p-TBK1/TBK1, p-IRF3/IRF3, and p-IRF7/IRF7 in the kidneys (E), and the densitometric analysis was conducted to analyze Western-blot in (F-H). Each lane represents samples from different mice. (A-G, H) n=6 mice/group. The results are representative of two independent experiments. Data are showed as means ± SEM. (C-D, F, H) Unpaired two-tailed Student’s t-test. (A-B, G) Mann-Whitney test. ns, not statistically significant. Supplemental Figure 3. The protein expression levels of relevant molecules in kidneys of uninfected mice. Uninfected WT and Aim2-/- mice were sacrificed and the kidneys were harvested. The protein expression level of inflammatory signaling pathways, inflammasome, type I interferon and apoptosis was evaluated by Western-blot. (A) The protein levels of p-IκB/IκB, p-ERK/ERK, p-JNK/JNK, p-p38/p-38; (B) The protein levels of caspase-1 (Casp1) and Gasdermin-D (GSDMD); (C) the protein levels of p-TBK1/TBK1, p-IRF3/IRF3, and p-IRF7/IRF7; (D) The protein levels of cleaved-caspase3/caspase 3 and cleaved-caspase 7/caspase 7. Each lane represents samples from different mice. Supplemental Figure 4. Aim2 gene expression in macrophages/dendritic cells. The expression level of Aim2 was evaluated in WT BMDMs, BMDCs and peritoneal macrophages of WT mice by RT-qPCR. (A) The level of Aim2 was evaluated with Aim2 Ct value minus Actb Ct value.Supplemental Figure 5. AIM2 deficiency does not affect phagocytosis and killing of C. albicans by macrophages. BMDMs obtained from both WT and Aim2-/- mice were subjected to stimulation with live C. albicans (MOI = 10) for a duration of 2 hours (for phagocytosis) or 24 hours (for killing). The in vitro phagocytic and killing capacities of the BMDMs were quantified as (A) and (B), respectively. The results are representative of three independent experiments. Data are shown as means±SEM. (A-B) Unpaired two-tailed Student’s t-test. ns, not statistically significant.Supplemental Figure 6. Apoptosis induction is reduced in Aim2-/- BMDMs by C. albicans DNA transfection. Bone marrow-derived macrophages (BMDMs) from WT and Aim2-/- mice were infected with live C. albicans (MOI = 5) or transfected with C. albicans DNA (2.5 μg) ，poly (dA: dT) (2.5 μg) or empty vector group PEI and Lyo Vector for 8 h, and cell lysates were analyzed for caspase-3/7 activation by Western-blot (A). The results are representative of two independent experiments. Supplemental Figure 7. Apoptosis is increased in macrophages after C. albicans infection. WT BMDMs were either uninfected or infected with live C. albicans for 8 h at MOI 5. The slides were stained with SYTOX dye (green) and anti-F4/80 (red), analyzed by confocal microcopy. Representative images for each were shown. Cells were counterstained with DAPI (blue). Images were representative of two independent experiments. Scale bar = 20um. Supplemental Figure 8. AIM2 inhibits Akt phosphorylation in vivo and vitro after C. albicans infection. (A-B) WT mice were either treated with PBS (Control) or C. albicans (Infected) (2×105 CFU) and sacrificed on 3rd day post-infection. and the kidney tissues were collected for Akt analysis. (A) The protein levels of p-AKT/AKT in kidneys. Each lane represents samples from different mice. (B) Densitometric analysis of Western-blot results in (A). (C-F) Aim2fl/fl and Aim2fl/fl; CD11c-cre or Aim2fl/fl; Lyz-cre mice were subjected to intravenous infection with C. albicans (2×105 CFU) and sacrificed on the third day post-infection. The protein levels of p-AKT/AKT in kidneys of Aim2fl/fl; CD11c-cre mice (C-D) or of Aim2fl/fl; Lyz-cre mice (E-F). Each lane represents samples from different mice. The results are representative of two independent experiments. BMDMs from WT and Aim2-/- mice were infected with live C. albicans (MOI = 5) or transfected with C. albicans DNA (2.5 μg) ，poly (dA: dT) (2.5 μg) or empty vector group PEI and Lyo Vector for 30 min, and cell lysates were analyzed for Akt phosphorylation by Western-blot (G). The results are representative of two independent experiments. Data are shown as means±SEM. (B, D, F) Unpaired two-tailed Student’s t-test. *P < 0.05, ***P < 0.001, and ns, not statistically significant. Supplemental Figure 9. Schematic diagram of the working model. After C. albicans infection, AIM2 in macrophages recognizes C. albicans DNA and subsequently inhibits AKT phosphorylation and enhances cell apoptosis, thereby resulting in the evasion and proliferation of C. albicans.

Supplementary file2 (PDF 304 KB)

## Data Availability

Data will be made available upon reasonable request.
