# Supplementary material for: AIM2 enhances Candida albicans infection through promoting macrophage apoptosis via AKT signaling
Source: Cell Mol Life Sci. 2024 Jun 25;81(1):280. doi: 10.1007/s00018-024-05326-9 (PMC11335202; doi:10.1007/s00018-024-05326-9)
Supplement: Supplementary file 1 — Supplementary file1 (PDF 689 KB) Supplemental Figure 1. The validation of BMDMs/BMDCs. The single-cell suspensions of WT BMDMs and BMDCs were staining with antibodies and analyzed by flow cytometry. (A) Gating strategy of flow cytometry and the proportion of F4/80+ cells. (B) Gating strategy of flow cytometry and the proportion of CD11c+cells. Supplemental Figure 2. The resistance of Aim2-/- mice to C. albicans infection is not associated with the production of type I interferons. WT and Aim2-/- mice were subjected to C. albicans infection, and kidney, sera were collected at 3 days post-infection. The gene and protein expression levels of IFN-α and IFN-β were evaluated using RT-qPCR (A-B) and ELISA (C-D), respectively. Western-blot analysis was performed to determine the protein levels of p-TBK1/TBK1, p-IRF3/IRF3, and p-IRF7/IRF7 in the kidneys (E), and the densitometric analysis was conducted to analyze Western-blot in (F-H). Each lane represents samples from different mice. (A-G, H) n=6 mice/group. The results are representative of two independent experiments. Data are showed as means ± SEM. (C-D, F, H) Unpaired two-tailed Student’s t-test. (A-B, G) Mann-Whitney test. ns, not statistically significant. Supplemental Figure 3. The protein expression levels of relevant molecules in kidneys of uninfected mice. Uninfected WT and Aim2-/- mice were sacrificed and the kidneys were harvested. The protein expression level of inflammatory signaling pathways, inflammasome, type I interferon and apoptosis was evaluated by Western-blot. (A) The protein levels of p-IκB/IκB, p-ERK/ERK, p-JNK/JNK, p-p38/p-38; (B) The protein levels of caspase-1 (Casp1) and Gasdermin-D (GSDMD); (C) the protein levels of p-TBK1/TBK1, p-IRF3/IRF3, and p-IRF7/IRF7; (D) The protein levels of cleaved-caspase3/caspase 3 and cleaved-caspase 7/caspase 7. Each lane represents samples from different mice. Supplemental Figure 4. Aim2 gene expression in macrophages/dendritic cells. The expression level of Aim2 [file 18_2024_5326_MOESM1_ESM.pdf]

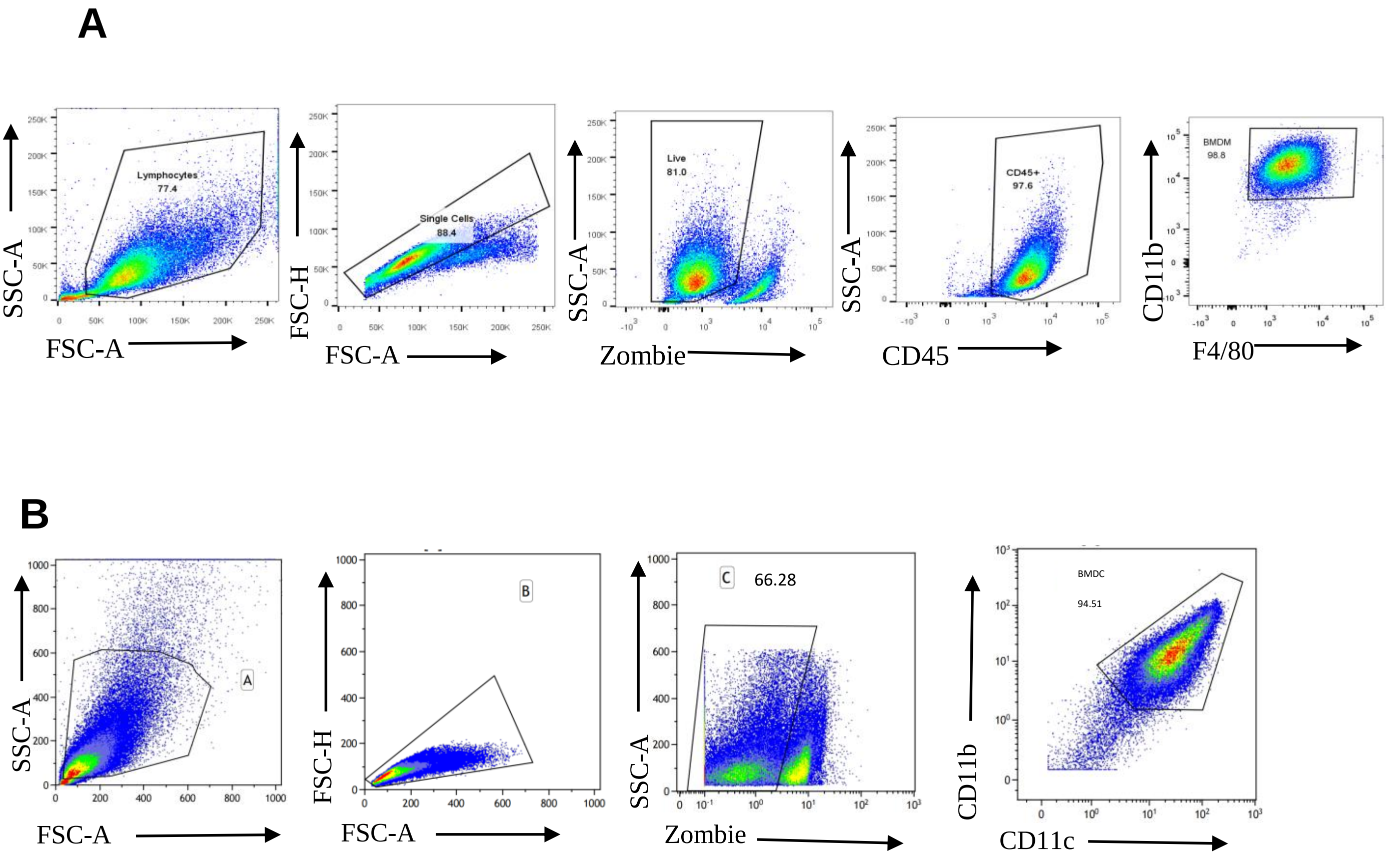

Supplementary Fig.1

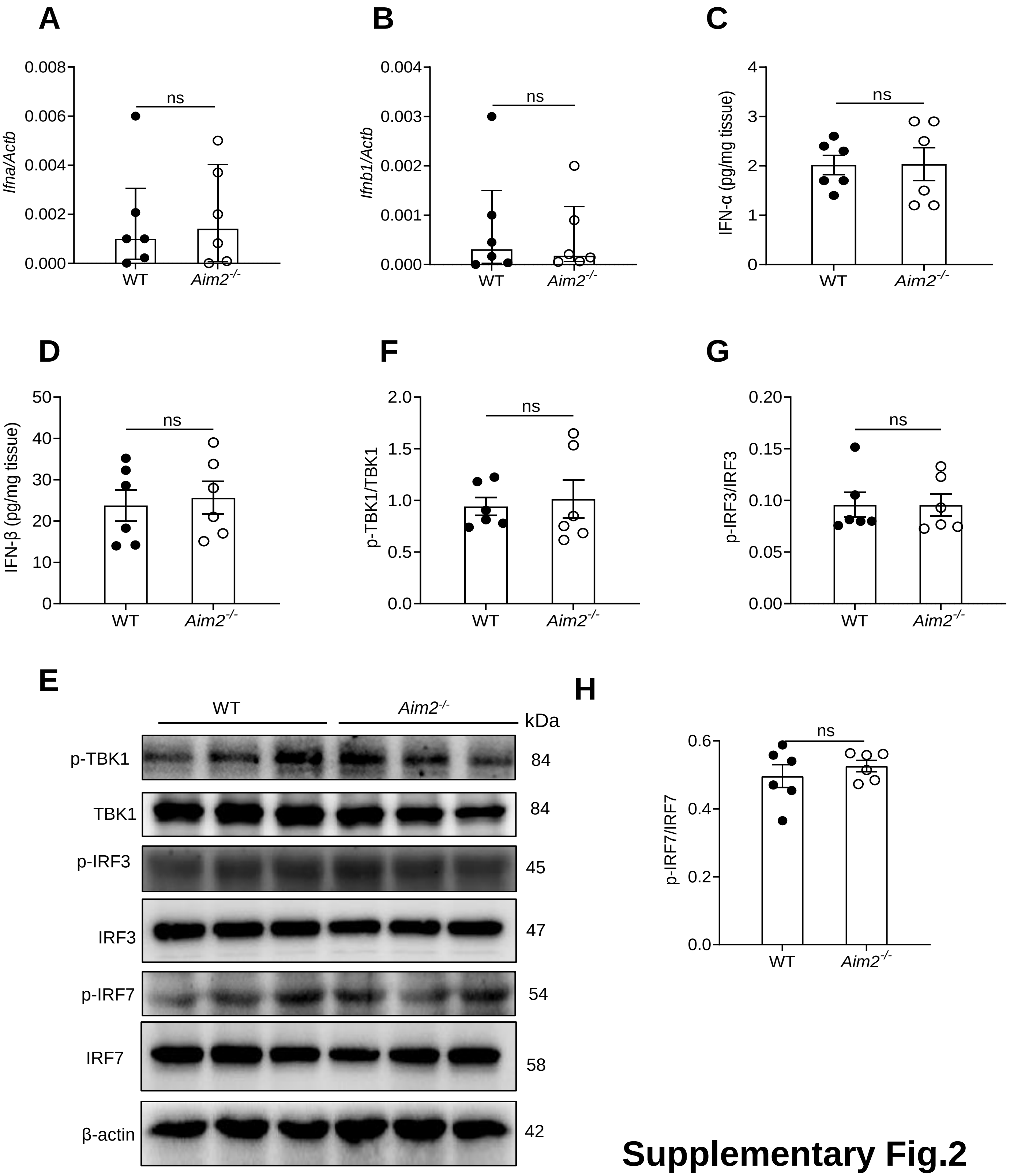

Supplementary Fig.2

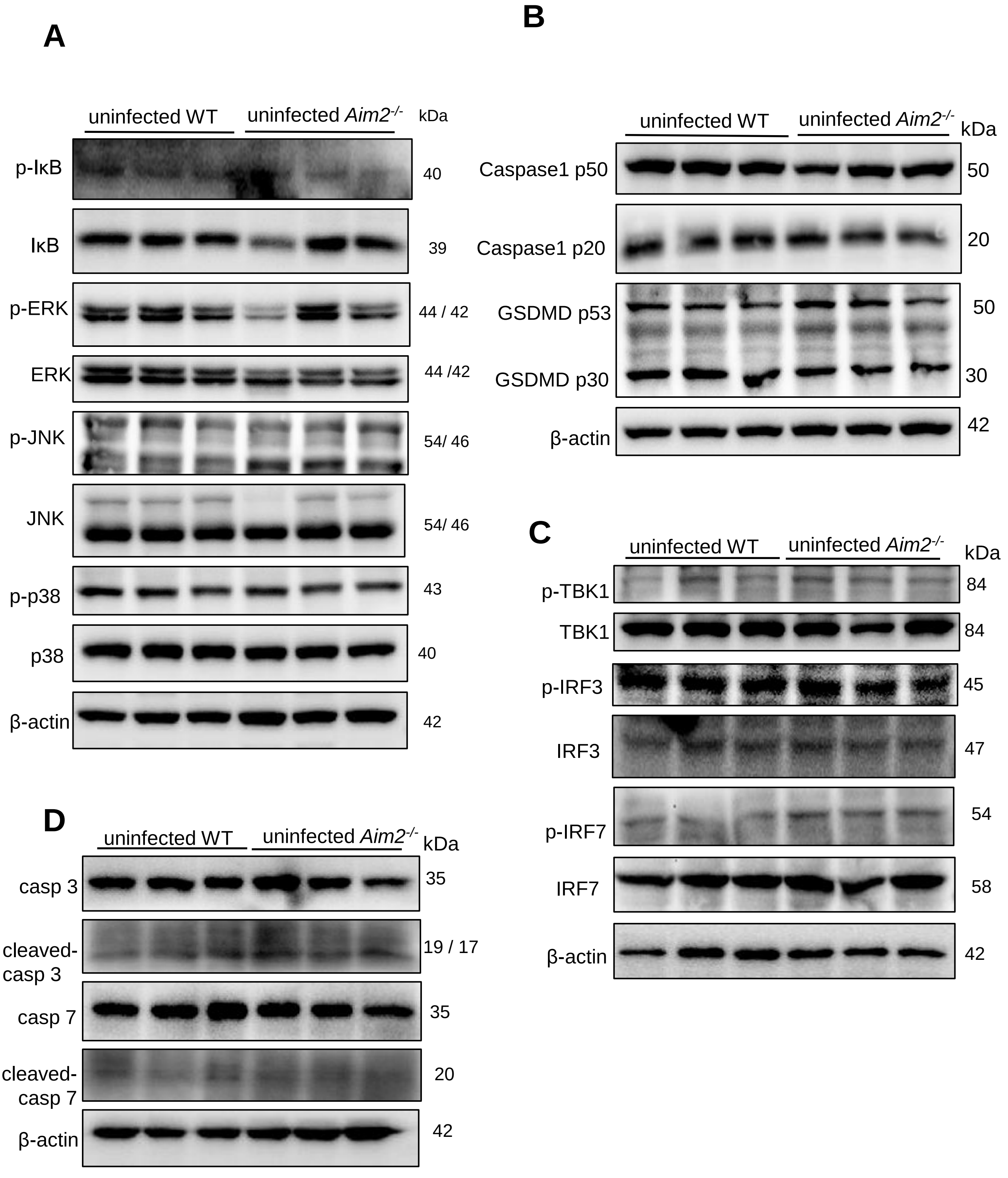

Supplementary Fig.3

A

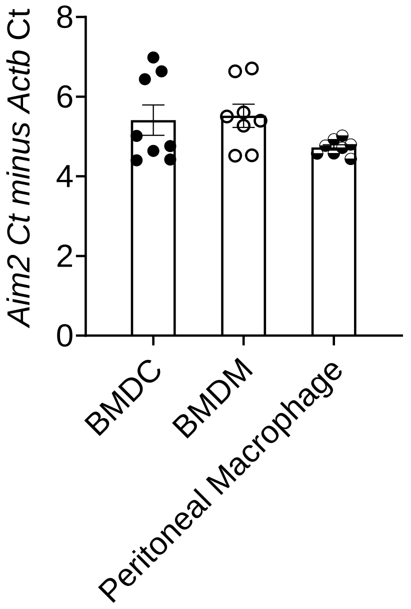

Supplementary Fig.4

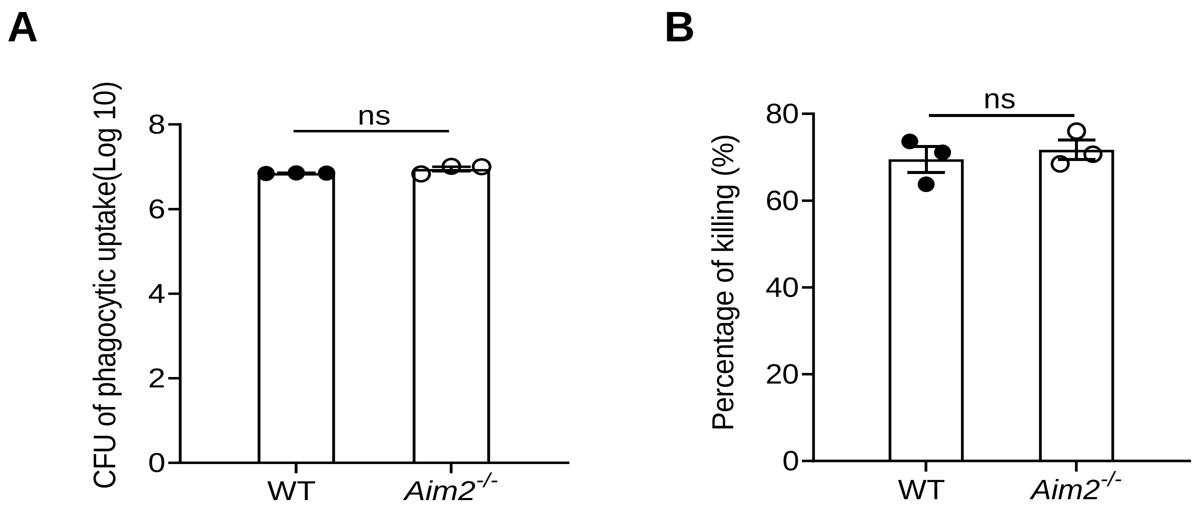

Supplementary Fig.5

A

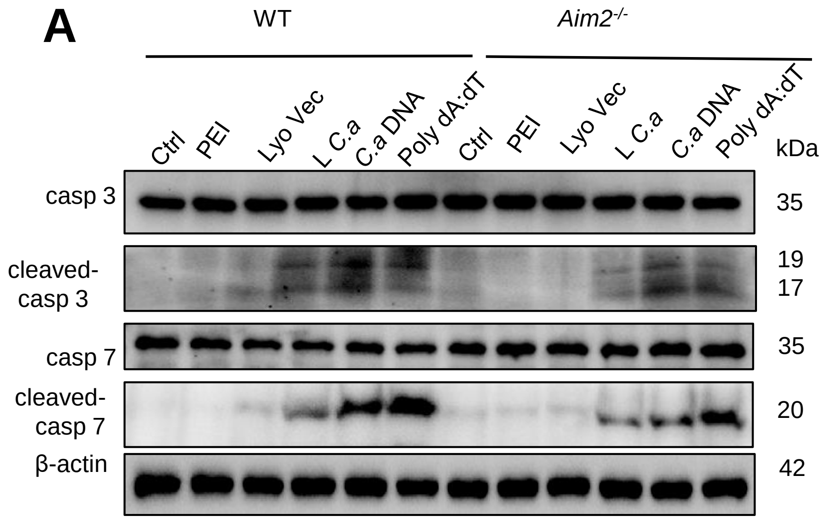

Supplementary Fig.6

A

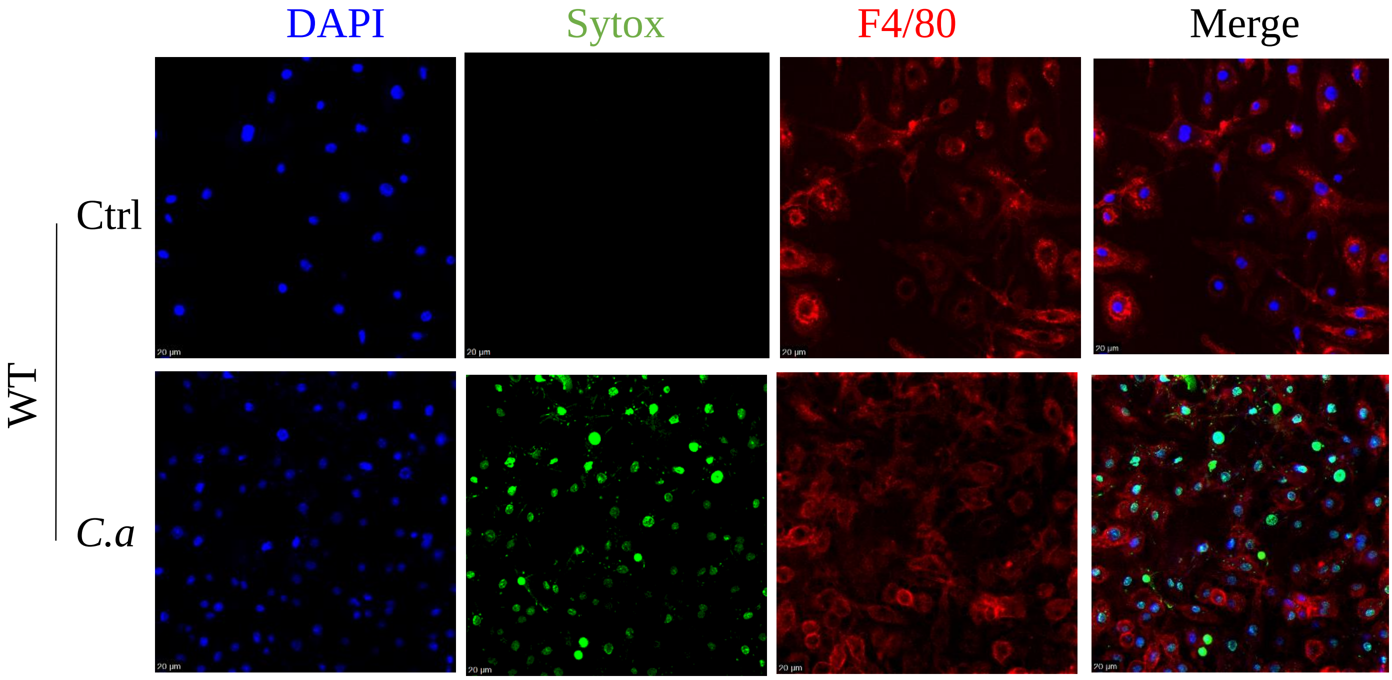

Supplementary Fig.7

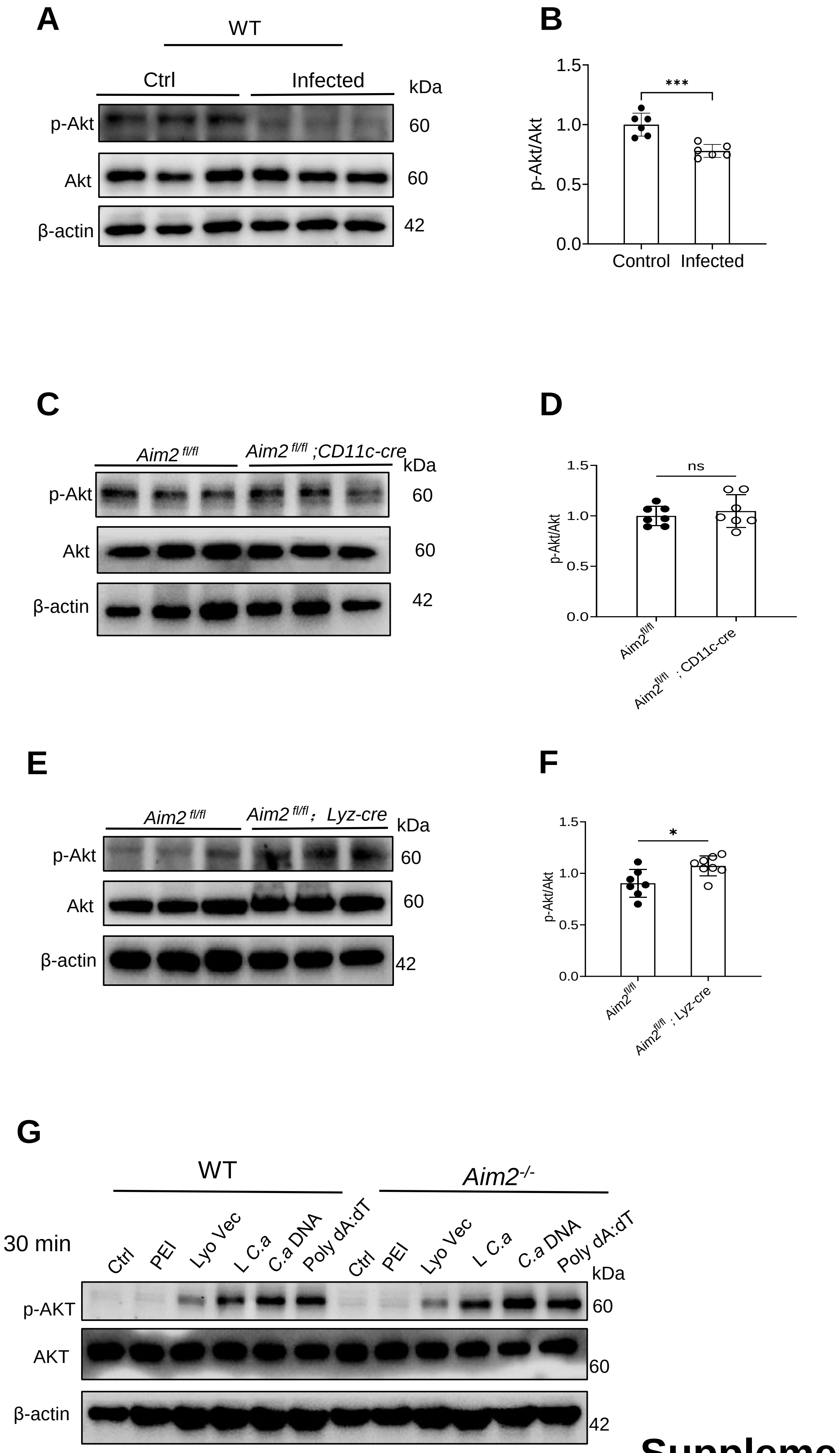

Supplementary Fig.8

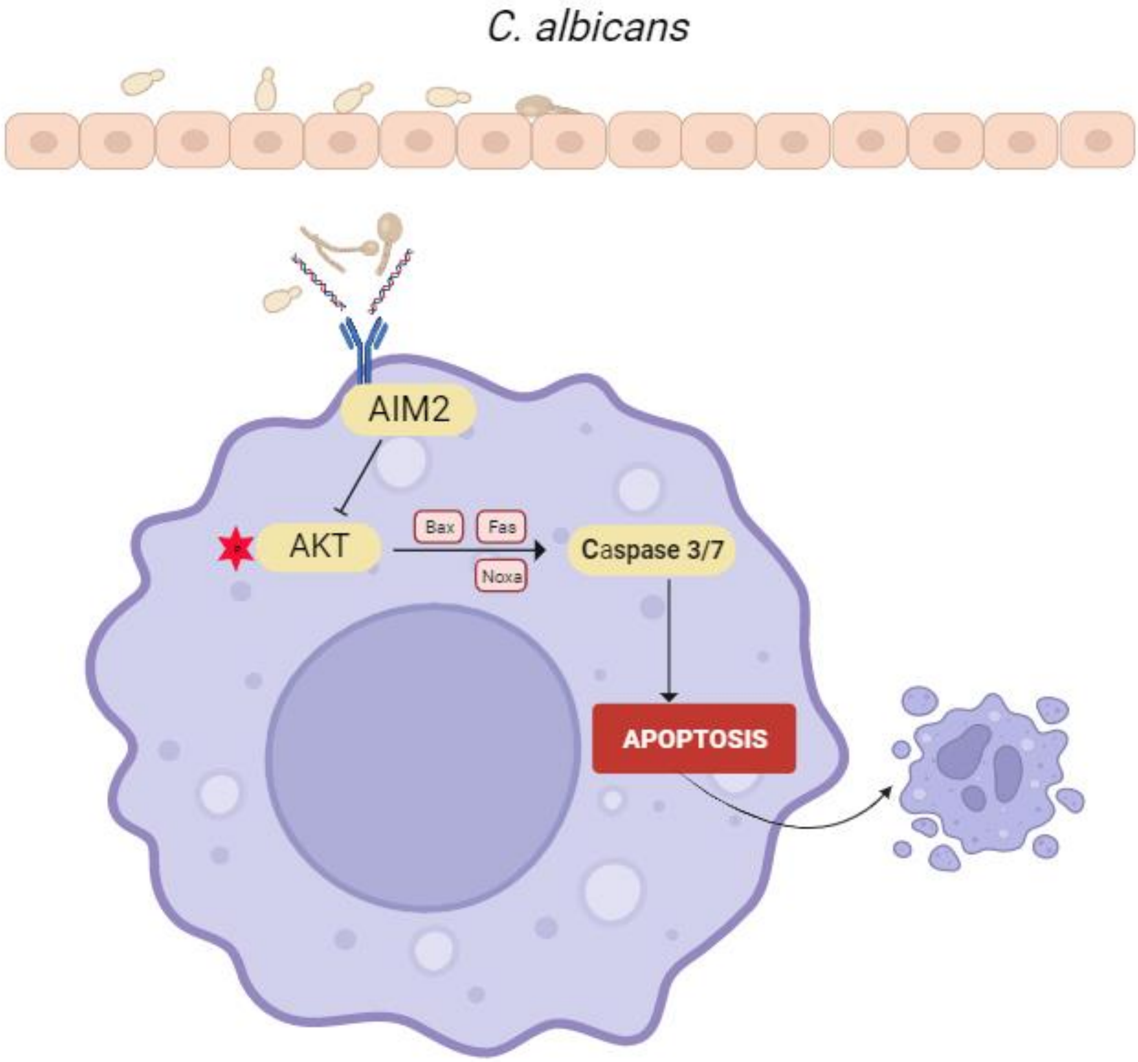

Supplementary Fig.9
