# Supplementary material for: AIM2 enhances Candida albicans infection through promoting macrophage apoptosis via AKT signaling
Source: Cell Mol Life Sci. 2024 Jun 25;81(1):280. doi: 10.1007/s00018-024-05326-9 (PMC11335202; doi:10.1007/s00018-024-05326-9)
Supplement: Supplementary file 2 — Supplementary file2 (PDF 304 KB) [file 18_2024_5326_MOESM2_ESM.pdf]

Supplementary Table 1. Primers used in this study

Gene	Primer	sequence 5' to 3'
Tnfα	Forward	CCACCACGCTCTTCTGTCTACTG
	Reverse	GGGCTACGGGCTTGTCACTC
Il6	Forward	AAGACAAAGCCAGAGTCATTCAGAG
	Reverse	GTTGGATGGTCTTGGTCCTTAGC
Kc	Forward	CTGGGATTCACCTCAAGAACATC
	Reverse	CAGGGTCAAGGCAAGCCTC
Mcp-1	Forward	CTTCTGGGCCTGCTGTTCA
	Reverse	CCAGCCTACTCATTGGATCA
Ifnα	Forward	CTGTGCTTTCCTGATGGT
	Reverse	GCTTGGTGGTTTGCTACGAC
Ifnb	Forward	CCATGGGAATCAACTATAAGCAGCTC
	Reverse	GAAGCTTGTTTTGGAAGTTTCTGGT
Bcl-2	Forward	ATGCCTTTGTGGAACTATATGGC
	Reverse	GGTATGCACCCAGAGTGATGC
Bax	Forward	TGAAGACAGGGGCCTTTTTG
	Reverse	AATTCGCCGGAGACACTCG
Fas	Forward	TATCAAGGAGGCCCATTTTGC
	Reverse	TGTTTCCACTTCTAAACCATGCT
Noxa	Forward	GCAGAGCTACCACCTGAGTTC
	Reverse	CTTTTGCGACTTCCCAGGCA
Bak1	Forward	CAACCCCGAGATGGACAACTT
	Reverse	CGTAGCGCCGGTTAATATCAT
Bad	Forward	AAGTCCGATCCCGGAATCC
	Reverse	GCTCACTCGGCTCAAACTCT
Mcl-1	Forward	AAAGGCGGCTGCATAAGTC
	Reverse	TGGCGGTATAGGTCGTCCTC
Aim2	Forward	GAT TCA AAG TGC AGG TGC GG
	Reverse	TCT GAG GCT TAG CTT GAG GAC
Gapdh	Forward	AGGTCGGTGTGAACGGATTTG
	Reverse	TGTAGACCATGTAGTTGAGGTCA
Actb	Forward	CCAACCGTGAAAAGATGACC
	Reverse	ACCAGAGGCATACAGGGACA

Supplementary Table 2. ELISA kits used in this study

Antibodies	Identifier	Distributor
IL-6	#02446M1	Jingmei Biotechnology
TNF -α	#02415M1	Jingmei Biotechnology
KC	#03093M1	Jingmei Biotechnology
MCP-1	#02365M1	Jingmei Biotechnology
IFN-α	#02649M1	Jingmei Biotechnology
IFN-β	#11796M1	Jingmei Biotechnology
IL-18	#02452M1	Jingmei Biotechnology
IL-1β	#02323M1	Jingmei Biotechnology

Supplementary Table 3. Antibodies used for Western-blot in this study

Antibodies	Identifier	Distributor
p-IRF3	# 29047	Cell signaling
IRF3	# ab68481	Abcam
p-IRF7	#PA5-106184	Invitrogen
IRF7	#PA5-102832	Invitrogen
p-TBK1	#5483	Cell signaling
TBK1	#ab109735	Abcam
p-IκB	#2859	Cell signaling
IκB	#9242	Cell signaling
p-JNK	#9251	Cell signaling
JNK	#9252	Cell signaling
p-p38	#9211	Cell signaling
p38	#8690	Cell signaling
p-ERK1/2	#9101	Cell signaling
ERK1/2	#4695	Cell signaling
Caspase-1	#AG-20B-0042-C100	AdipoGen
Caspase-3	#9665	Cell signaling
Cleaved Caspase-3(Asp175)	#9661	Cell signaling
Caspase-7	#9492	Cell signaling
Cleaved Caspase-7	#8438	Cell signaling
Bcl-2	#3498	Cell signaling
Bax	#2722s	Cell signaling
AKT	#4691	Cell signaling
P-AKT	#9271	Cell signaling
GSDMD	ab209845	Abcam
β- actin	#A1978	Sigma

Supplementary Table 4. Antibodies used for Flow cytometry in this study

Antibodies	Identifier	Distributor
anti-mouse CD16/32	156604	Biolegend
APC/ Cyanine7-Zombie	423105	Biolegend
PE/Cyanine7 anti-mouseCD45	157205	Biolegend
APC-anti-mouse CD11b	101211	Biolegend
PE- anti-mouse F4/80	123109	Biolegend
PE- anti-mouse CD11c	117307	Biolegend

Supplementary Table 5. GO term enrichment of AIM2 gene for *C. albicans* response

GSE database	Go Term	PValue	Bonferroni
GSE42606	GO:0051607~defense response to virus	9.00E-41	1.69E-37
	GO:0006954~inflammatory response	1.19E-26	2.24E-23
	GO:0006955~immune response	7.65E-26	1.44E-22
	GO:0045087~innate immune response	5.73E-22	1.08E-18
	GO:0050729~positive regulation of inflammatory response	1.21E-10	2.28E-07
	GO:0042802~identical protein binding	4.35E-10	2.26E-07
	GO:0005515~protein binding	1.47E-09	7.66E-07
	GO:0006915~apoptotic process	2.73E-09	5.12E-06
	GO:0032731~positive regulation of interleukin-1 beta production	8.38E-09	1.57E-05
	GO:0051092~positive regulation of NF-kappaB transcription factor activity	1.19E-07	2.23E-04
	GO:0035458~cellular response to interferon-beta	9.47E-07	0.00177578
	GO:0070269~pyroptosis	3.18E-06	0.005949265
	GO:0005829~cytosol	3.68E-05	0.010313014
GSE69723	GO:0006954~inflammatory response	2.05E-12	5.20E-09
	GO:0051607~defense response to virus	3.79E-09	9.59E-06
	GO:0045087~innate immune response	1.13E-06	0.002868807
GSE162476	GO:0006954~inflammatory response	6.95E-40	3.79E-36
	GO:0006955~immune response	1.54E-26	8.42E-23
	GO:0051607~defense response to virus	8.37E-24	4.56E-20
	GO:0045087~innate immune response	5.48E-20	2.98E-16

	GO:0050729~positive regulation of inflammatory response	6.14E-15	3.33E-11
	GO:0005515~protein binding	5.99E-14	9.00E-11
	GO:0032731~positive regulation of i nterleukin-1 beta production	4.47E-12	2.44E-08
	GO:0042802~identical protein binding	1.03E-09	1.55E-06
	GO:0051092~positive regulation of NF-kappaB transcription factor activity	1.26E-08	6.86E-05
	GO:0038187~pattern recognition receptor activity	8.17E-08	1.23E-04
	GO:0006915~apoptotic process	1.37E-07	7.44E-04
	GO:0005737~cytoplasm	2.01E-05	0.014171346

The significantly enriched GO terms ordered for p-value with Bonferroni test were shown. $P < 0.05$ was considered statistically significant.
